# MicroRNA-100-5p and microRNA-298-5p released from apoptotic cortical neurons are endogenous Toll-like receptor 7/8 ligands that contribute to neurodegeneration

**DOI:** 10.1186/s13024-021-00498-5

**Published:** 2021-11-27

**Authors:** Thomas Wallach, Zoé J. Mossmann, Michal Szczepek, Max Wetzel, Rui Machado, Martin Raden, Milad Miladi, Gunnar Kleinau, Christina Krüger, Paul Dembny, Drew Adler, Yuanyuan Zhai, Victor Kumbol, Omar Dzaye, Jutta Schüler, Matthias Futschik, Rolf Backofen, Patrick Scheerer, Seija Lehnardt

**Affiliations:** 1 Institute of Cell Biology and Neurobiology, Charité – Universitätsmedizin Berlin, corporate member of Freie Universität Berlin, Humboldt-Universität zu Berlin, and Berlin Institute of Health, 10117 Berlin, Germany; 2 Institute for Medical Physics and Biophysics, Group Protein X-ray Crystallography & Signal Transduction, Charité – Universitätsmedizin Berlin, corporate member of Freie Universität Berlin, Humboldt-Universität zu Berlin, and Berlin Institute of Health, 10117 Berlin, Germany; 3 Department of Biomedical Sciences and Medicine, University of Algarve, 8005-139 Faro, Portugal; 4 Bioinformatics, Department of Computer Science, Albert-Ludwigs-University Freiburg, Freiburg, Germany; 5 Department of Radiology, Charité – Universitätsmedizin Berlin, corporate member of Freie Universität Berlin, Humboldt-Universität zu Berlin, and Berlin Institute of Health, 10117 Berlin, Germany; 6 School of Biomedical Sciences, Faculty of Health, University of Plymouth, Plymouth, PL6 8BU UK; 7 MRC London Institute of Medical Sciences (LMS), Faculty of Medicine, Imperial College London, London, W12 0NN UK; 8 German Centre for Cardiovascular Research, partner site Berlin, Berlin, Germany; 9 Department of Neurology, Charité – Universitätsmedizin Berlin, corporate member of Freie Universität Berlin, Humboldt-Universität zu Berlin, and Berlin Institute of Health, 10117 Berlin, Germany

**Keywords:** Extracellular microRNAs, Endogenous Toll-like receptor ligands, Cortical neurons, Neuronal apoptosis, Microglia, Neurodegeneration, miRNA microarray

## Abstract

**Background:**

MicroRNA (miRNA) expression in the brain is altered in neurodegenerative diseases. Recent studies demonstrated that selected miRNAs conventionally regulating gene expression at the post-transcriptional level can act extracellularly as signaling molecules. The identity of miRNA species serving as membrane receptor ligands involved in neuronal apoptosis in the central nervous system (CNS), as well as the miRNAs’ sequence and structure required for this mode of action remained largely unresolved.

**Methods:**

Using a microarray-based screening approach we analyzed apoptotic cortical neurons of C56BL/6 mice and their supernatant with respect to alterations in miRNA expression/presence. HEK-Blue Toll-like receptor (TLR) 7/8 reporter cells, primary microglia and macrophages derived from human and mouse were employed to test the potential of the identified miRNAs released from apoptotic neurons to serve as signaling molecules for the RNA-sensing receptors. Biophysical and bioinformatical approaches, as well as immunoassays and sequential microscopy were used to analyze the interaction between candidate miRNA and TLR. Immunocytochemical and -histochemical analyses of murine CNS cultures and adult mice intrathecally injected with miRNAs, respectively, were performed to evaluate the impact of miRNA-induced TLR activation on neuronal survival and microglial activation.

**Results:**

We identified a specific pattern of miRNAs released from apoptotic cortical neurons that activate TLR7 and/or TLR8, depending on sequence and species. Exposure of microglia and macrophages to certain miRNA classes released from apoptotic neurons resulted in the sequence-specific production of distinct cytokines/chemokines and increased phagocytic activity. Out of those miRNAs miR-100-5p and miR-298-5p, which have consistently been linked to neurodegenerative diseases, entered microglia, located to their endosomes, and directly bound to human TLR8. The miRNA-TLR interaction required novel sequence features, but no specific structure formation of mature miRNA. As a consequence of miR-100-5p- and miR-298-5p-induced TLR activation, cortical neurons underwent cell-autonomous apoptosis. Presence of miR-100-5p and miR-298-5p in cerebrospinal fluid led to neurodegeneration and microglial accumulation in the murine cerebral cortex through TLR7 signaling.

**Conclusion:**

Our data demonstrate that specific miRNAs are released from apoptotic cortical neurons, serve as endogenous TLR7/8 ligands, and thereby trigger further neuronal apoptosis in the CNS. Our findings underline the recently discovered role of miRNAs as extracellular signaling molecules, particularly in the context of neurodegeneration.

**Supplementary Information:**

The online version contains supplementary material available at 10.1186/s13024-021-00498-5.

## Background

Neurodegenerative disorders are characterized by progressive neuronal injury and loss. Innate immune receptors such as Toll-like receptors (TLRs) can contribute to such central nervous system (CNS) injury by triggering an inflammatory response, but also by mediating cell-autonomous damage [1]. To date, 10 TLRs were identified in humans and 13 in mice. TLRs are transmembrane proteins composed of an extracellular domain dominated by leucine-rich repeats responsible for ligand binding, and a cytoplasmic Toll/IL-1 receptor domain necessary for binding of intracellular adaptor molecules. TLRs are activated by highly conserved pathogen-associated molecular patterns but also by host-derived molecules, which leak into the extracellular space upon cellular injury and are referred to as danger-associated molecular patterns (DAMPs) [2]. DAMP proteins such as α-synuclein [3] and amyloid-β [4] contribute to pathological processes in Parkinson’s and Alzheimer’s disease (AD), respectively. In contrast, the role of RNA species functioning as DAMPs in CNS injury is less well characterized. TLR activation induced by both DAMPs and pathogens results in receptor dimerization and recruitment of adaptor molecules, leading to the translocation of transcription factors, such as NF-κB, into the nucleus, and the subsequent release of inflammatory mediators [2]. Some TLRs can mediate injury in a cell-autonomous fashion, particularly in the brain [5–7].

TLR7 and TLR8 are primarily localized to the endosomal compartment of immune cells [2], such as microglia, the resident immune cells in the CNS, but were also detected in neurons [6, 8]. Both receptors are phylogenetically and structurally closely related and are activated by GU-rich and AU-rich sequences present in single-stranded RNA (ssRNA) [9–11]. Such sequences are predominantly present in viral RNA including human immunodeficiency virus (HIV) and influenza virus [10, 12]. Certain host-derived ssRNAs, in particular microRNAs (miRNAs), also contain distinct GU-rich sequence motifs, which are crucial for receptor activation [6]. miRNAs are noncoding ssRNAs with a length of 18 to 22 nucleotides. They are evolutionarily highly conserved across all phyla including humans. In their canonical function, miRNAs regulate gene expression by binding to the 3′ untranslated regions of mRNA through imperfect base pairing, facilitating protein translation repression, mRNA deadenylation, and subsequent degradation. As perfect complementary base pairing is not  required, a single miRNA can bind to the 3′ UTR of different mRNAs, while a single mRNA may be targeted by multiple miRNAs [13]. In addition, a novel role of miRNAs as extracellular signaling molecules has been recently discovered [6, 14, 15]. Defined pathological conditions, such as malignancy and cellular stress, result in the release of a specific signature of miRNAs, which are associated with extracellular vesicles or bound to carrier proteins, such as AGO2, which is part of the RNA-induced silencing complex [16]. For example, *let-7b* miRNA was detected extracellularly in the injured CNS [6]. Moreover, the expression profile of numerous miRNAs is altered in neurodegenerative diseases, including AD, multiple sclerosis, and amyotrophic lateral sclerosis [17–20]. However, the functional relevance of such changes for the underlying CNS pathologies is poorly understood.

Here, we sought to systematically identify and determine the role of miRNAs as immunomodulatory signaling molecules in neurodegeneration. Using a GeneChip miRNA array approach, we found that cortical neurons release a specific signature of miRNAs upon induction of apoptosis. Twelve miRNAs were selected, six of which activated TLR7 and/or TLR8. In particular, miR-100-5p and miR-298-5p induced an inflammatory response through activation of microglia and macrophages. To gain a deeper understanding of the direct interaction between these miRNAs and TLRs, we applied imaging-based, biophysical, and bioinformatical methods, thereby visualizing the miRNAs’ entry into a cell, determining miRNA ligand-receptor binding affinity, and characterizing the miRNA ligands’ sequence features. The functional relevance of miR-100-5p and miR-298-5p acting as endogenous TLR7/8 ligands in the CNS was elaborated in mouse models of neuronal injury in vitro and in vivo.

## Methods

### Mice and cell lines

C57BL/6 and *Tlr7*^*−/−*^ mice were bred at the FEM, Charité – Universitätsmedizin Berlin, Germany. *Tlr7*^*−/−*^ mice were generously provided by S. Akira (Osaka University, Osaka, Japan). Animals were maintained according to the guidelines of the committee for animal care. All animal procedures were approved by the Landesamt für Gesundheit und Soziales (LAGeSo) Berlin, Germany. HEK-Blue™ Secreted Embryonic Alkaline Phosphatase (SEAP) cells expressing human TLR7 or human TLR8, as well as the respective control cell lines HEK-Blue™ Null1-k and Null1 (Invivogen, San Diego, CA, USA) were cultured in Dulbecco’s modified Eagle’s medium (DMEM; Invitrogen #41965062, Carlsbad, CA, USA). DMEM was supplemented with 10% heat-inactivated fetal calf serum (FCS, Gibco #10082–147, Thermo Fisher Scientific, Waltham, MA, USA) and penicillin (100 U/ml)/streptomycin (100 μg/ml; both obtained from Gibco #15140–122, Thermo Fisher Scientific, Waltham, MA, USA). THP-1 cells were kindly provided by Dr. Elisabeth Kowenz-Leutz, Max Delbrück Center for Molecular Medicine, Berlin, Germany, and cultured in RPMI-1640 medium (Gibco # A1451701, Thermo Fisher Scientific, Waltham, MA, USA), supplemented with 0.05 mM 2-mercaptoethanol, 10% (v/v) heat-inactivated FCS, and penicillin (100 U/ml)/streptomycin (100 μg/ml, both obtained from Gibco #15140–122, Thermo Fisher Scientific, Waltham, MA, USA). Cells were cultured at 37 °C in humidified air with 5% (v/v) CO_2_.

### Primary cultures of cortical neurons

Primary cultures of cortical neurons were generated from forebrains of embryonic (E) 17.5 mice, as previously described [21]. Briefly, meninges, superficial blood vessels, and cerebellum were removed from the cortices. Cortices were then homogenized and incubated with 0.5 ml Trypsin (0.5%) for 20 min at 37 °C. Trypsin reaction was stopped with heat-inactivated FCS (Gibco #10082–147, Thermo Fisher Scientific, Waltham, MA, USA). 100 μl DNase (Roche #ROD 1284932, Basel, Switzerland) were added. After several washing steps, cell suspension was centrifuged at 300 rpm at 4 °C for 4 min. Supernatant was collected and centrifuged at 1200 rpm at 4 °C for further 5 min. Pellets were resuspended in Neurobasal Medium (Gibco #21103–049, Thermo Fisher Scientific, Waltham, MA, USA) supplemented with 1% L-Glutamine (Gibco #25030–024 Thermo Fisher Scientific, Waltham, MA, USA), 1% penicillin/streptomycin (Gibco #15140–122, Thermo Fisher Scientific, Waltham, MA, USA), and 2% B27 supplement (Gibco #17504–044, Thermo Fisher Scientific, Waltham, MA, USA). Neurons were seeded into a 24-well plate at a density of 5 × 10^5^ cells/well and incubated for 3 d at 37 °C in humidified air with 5% (v/v) CO_2_, before starting the experiment.

### Primary cultures of microglia

Primary cell cultures of microglia were generated as previously described [22]. Briefly, microglia were isolated from mouse brains on postnatal day (P) 1–4. Meninges, superficial blood vessels, and cerebellum were removed from cortices. Cortices were then homogenized with 3 ml Trypsin (2.5%; Gibco #15090–046, Thermo Fisher Scientific, Waltham, MA, USA) for 25 min at 37 °C. Trypsin reaction was stopped with FCS (Gibco #10082–147, Thermo Fisher Scientific, Waltham, MA, USA). 100 μl DNase (Roche #ROD 1284932, Basel, Switzerland) were added. Cell suspension was centrifuged at 1200 rpm at 4 °C for 5 min. Pellets were resuspended in DMEM (Invitrogen #41965062, Carlsbad, CA, USA) supplemented with 10% FCS (Gibco #10082–147, Thermo Fisher Scientific, Waltham, MA, USA) and 1% penicillin/streptomycin (Gibco #15140–122, Thermo Fisher Scientific, Waltham, MA, USA), mechanically disassociated, and passed through a 70-μm-cell strainer. Microglia were grown in T75 flasks for 10–14 d in 12 ml DMEM (Invitrogen #41965062, Carlsbad, CA, USA) at 37 °C in humidified air with 5% (v/v) CO_2_.

### Co-cultures of microglia and neurons

For microglia/neuron co-cultures, half of the medium of 500,000 neurons cultured in a 24-well plate was replaced by fresh DMEM containing 60,000 microglia at day 3 after plating. On the following day, cells were used for experiments.

### Induction of apoptosis in primary cortical neurons

Staurosporine (Merck #S6942, Kenilworth, NJ, USA) was added to Neurobasal Medium (Gibco #21103–049, Thermo Fisher Scientific, Waltham, MA, USA) to reach a final concentration of 1 μM. Whole medium from primary neuron cell cultures was removed and replaced by staurosporine-containing medium. Primary neuron cultures were incubated with staurosporine for 8 h.

### Western blot analysis

Neurons were homogenized in 500 μl RIPA buffer (phosphate-buffered saline (PBS) with 1% IGEPAL CA-630, 0.5% Na-Deoxycholat, 0.1% SDS, 1:100 protease inhibitor #P8340, 1:100 phosphatase inhibitor #P2850, Sigma-Aldrich, Missouri, MO, USA). Protein concentration was determined using the Pierce BCA Protein Assay Kit (Thermo Fisher Scientific, #23227, Waltham, MA, USA). 50 μg of total protein were resolved by SDS-PAGE and then transferred to a nitrocellulose membrane followed by the incubation of primary and secondary antibodies according to the manufacturer’s protocol (active caspase-3 (1:1000), Cell Signaling Technology, #9661 Asp175, Danvers, MA, USA; anti-beta actin (0.5 μg/ml), Sigma Aldrich, #A1978-100UL, St. Louis, MO, USA; donkey anti-rabbit IgG-HRP #sc-2313; goat-anti-mouse IgG-HRP #sc-2005 Santa Cruz Biotechnology, Dallas, TX, USA).

### Small RNA extraction

miRNA extraction from conditioned medium and cortical neurons was performed as previously described [23, 24] using organic extraction followed by *mir*Vana™ isolation kit (Thermo Fisher Scientific #AM1560, Waltham, MA, USA) application. Briefly, 1 ml of cell-conditioned medium was used for organic total RNA extraction. 2 × 10^6^ cells grown on a 6-well plate were used for organic extraction from neurons. Cells were lysed prior to organic extraction. 700 μl of Trizol (Thermo Fisher Scientific #15596026, Waltham, MA, USA) were added to the sample, which was vortexed for 10 s, and subsequently was allowed to rest at room temperature for 5 min. 140 μl of chloroform were added. Samples were shaken for 15 s and subsequently centrifuged at 14,000 rpm, 4 °C for 15 min. Following centrifugation, the upper aqueous phase was transferred to a new tube. The miRNA-containing small RNA fraction was recovered using the *mir*Vana™ isolation kit (Thermo Fisher Scientific #AM1560, Waltham, MA, USA) according to the manufacturer’s protocol. In short, the aqueous phase was mixed with ethanol and passed through the filter cartridge by centrifugation. The filtrate was collected, mixed again with ethanol, and passed through a second filter cartridge by centrifugation. The flow-through was discarded, and the filter was washed with washing solution provided by the manufacturer. Finally, miRNA was eluted with nuclease-free water. RNA quantity and quality was determined using Nanodrop 2000c (Thermo Fisher Scientific, Waltham, MA, USA) and the Agilent 2100 Bioanalyzer system (Agilent Technologies, Santa Clara, CA, USA) using the Agilent 2100 Small RNA Kit/Chip Kit (Agilent Technologies, #5067–1548, Santa Clara, CA, USA).

### miRNA microarray

Affymetrix GeneChip miRNA 4.0 array (Thermo Fisher Scientific #902445, Waltham, MA, USA) harboring probes for 1908 mature mouse miRNAs were subjected to hybridization at the core facility *Labor für Funktionelle Genomforschung* (*LFGC*), Charité-Universitätsmedizin Berlin, Berlin, Germany.

### Microarray data analysis

Microarray data from the GeneChip miRNA 4.0 array (Thermo Fisher Scientific #902445, Waltham, MA, USA) were processed using the robust multi-array average (RMA) method on the R/Bioconductor platform [25]. Variability of RMA-processed data was visualized using principal components analysis (PCA) and density plots implemented in R. Separate PCA plots were produced for apoptotic neurons and conditioned medium with the respective controls displaying the overall variability in gene expression between samples. To verify whether the processed gene expression data were sufficiently normalized, density plots were generated. While PCA and density plots were generated based on the complete set of Affymetrix probe sets on the Gene-Chip (*n* = 36.353), subsequent analysis was restricted to miRNA probes that were specific for *Mus musculus.* Microarray TW16_231117_ MS_M3_2_2.cel was removed from this analysis due to insufficient results obtained by quality control. For differential expression analysis, the Bioconductor package *limma* [26] and multivariate empirical Bayes [27] statistics were applied providing Log2 Fold changes with corresponding Benjamini-Hochberg-adjusted *P* values. miRNAs expressed differentially were identified by performing the comparison of staurosporine-treated neurons and corresponding supernatant to the respective untreated neuron and medium control. A miRNA was considered as differentially expressed if it displayed an absolute Log2 Fold change > 1 and an adjusted *P* < 0.01. To generate a heat map of differential expression and an associated dendrogram, an in-house customized R programming script based on the package *ggplot2* was used [28]. The color scale in the heat map corresponds to the RMA-processed miRNA expression values after averaging over replicates. The microarray dataset was submitted to GEO (GSE165263) and will be provided upon publication.

### Gene ontology (GO) analysis of miRNAs present in neuronal supernatant

After obtaining 39 significantly enriched miRNAs in the supernatant of neurons through *limma* analysis [26] (see above), they were analysed regarding gene targeting. To this end, publicly available datasets that compile miRNA-mRNA interactions from 5 different online tools [29] were used. Four tools (PITA [30], microRNA.org [31], miRDB [32], and TargetScan [33]) contained computationally predicted miRNA-mRNA interactions, while one tool (MirTarBase [34]) contained experimentally validated miRNA-mRNA interaction data. Based on the integrated miRNA-mRNA data, gene targets were found for 35 out of 39 miRNAs. Next, Gene Ontology enrichment for biological processes of the targeted genes was performed using an in-house R script with functionalities of the GOstats package [35]. Significant of enrichment was assessed using the hypergeometric test, and *p*-values were adjusted using the Benjamini-Hochberg procedure. For 31 out of the identified 39 miRNAs, target genes could be linked to biological processes with statistical significance for enrichment (adjusted *P* < 0.01). Ultimately, selected relevant biological processes were gathered and graphically represented.

### Synthetic oligoribonucleotides

Oligoribonucleotides were modified with 5′ phosphorylation and phosphorothioate bonds in every base (Integrated DNA Technologies, Coralville, IA, USA). Fluorescent miRNA oligoribonucleotides were modified with 5′-Alexa488 (Integrated DNA Technologies, Coralville, IA, USA). Sequence information for experimentally tested miRNAs is provided in Table 1. The sequence of the mutated control oligoribonucleotide is enclosed in brackets (UGAGGUAGAAGGAUAUAAGGAU) [6].

**Table 1:** Full mature sequences, previously reported TLR-activating sequences (bold), and minimum free energy of selected miRNAs detected extracellularly enriched (Log2 Fold Changes; *P* values) upon neuronal apoptosis induction compared to control condition, as assessed by miRNA array

miRNA	Mature sequence	Log2 Fold Change	***P*** value	Minimum free energy [kcal/mol]
miR-151-5p	UCGAGGAGCUCACAGUCUAGU	5.28	9.60E-08	−0.6
miR-672-5p	UGAGGUUGGU**GUAC**UGUGUGUGA	4.83	5.94E-07	0
miR-674-5p	GCACUGAGAUGGGAGUGGUGUA	4.6	6.98E-06	−3.8
miR-361-5p	**UUAUC**AGAA**UCUC**CAGGG**GUAC**	4.47	0.007266	−0.1
let-7 g-5p	UGAGGUAGUA**GUUUGUAC**AGUU	4.46	2.84E-05	−0.5
miR-652-3p	AAUGGCGCCACUAGGG**UUGU**G	4.29	2.28E-06	−3.4
miR-342-5p	AGGGGUGC**UAUC**UGUGAUUGAG	4.28	4.39E-06	0
miR-100-5p	AACCCGUAGAUCCGAAC**UUGU**G	4.11	6.98E-05	0
miR-7020-5p	UGGGAUGGUGGAGAGGGUGACCAG	4.03	1.29E-07	−2.9
miR-128-3p	UCACAGUGAACCGG**UCUC**UUU	3.95	0.007777	0
miR-298-5p	GGCAGAGGAGGGC**UGUU**CUUCCC	3.55	2.28E-06	−5.5
miR-501-3p	AAUGCACCCGGGCAAGGAUUUG	3.65	4.60E-06	− 1.8

### HEK-blue TLR activation assay

Human TLR7 and human TLR8/NF-κB/Secreted Embryonic Alkaline Phosphatase (SEAP) reporter HEK293 cells were used for TLR activation assays. The parental control cell lines HEK-Blue Null-1 k and Null1, respectively, were used as control (all lines obtained from InvivoGen, San Diego, CA, USA). Cells were seeded into 96-well plates (5 × 10^4^/well). After 24 h, cells were incubated with the synthetic oligoribonucleotides or control oligonucleotide complexed to the transfection agent LyoVec (InvivoGen #LYEC-RNA, San Diego, CA, USA) according to the manufacturer’s instructions. Cells were stimulated with indicated agents dissolved in 90% HEK-Blue detection reagent (InvivoGen #hb-det2, San Diego, CA, USA) and 10% cell culture media. Each condition was performed in triplicate. The reporter protein SEAP was detected using the Varioskan Flash device (Thermo Fisher Scientific, Waltham, MA, USA) at a wavelength of OD 655 nm.

### THP-1 cell differentiation

THP-1 cells, a human monocyte-derived cell line, were cultured in RPMI-1640 medium (Gibco # A1451701, Thermo Fisher Scientific, Waltham, MA, USA) with 0.05 mM 2-mercaptoethanol (Merck, #M3148, Darmstadt, Germany), 10% heat-inactivated FCS (Gibco #10082–147, Thermo Fisher Scientific, Waltham, MA, USA), and penicillin (100 U/ml)/streptomycin (100 μg/ml; Gibco #15140–122, Thermo Fisher Scientific, Waltham, MA, USA). Four days before use, THP-1 cells were differentiated into macrophages by incubating the cells with 100 ng/ml phorbol 12-myristate 13-acetate (PMA, Merck, #P1585-PMA, Darmstadt, Germany) for 48 h. Cells were cultured for another 2 d in PMA-free medium and were grown under standard cell culture conditions.

### TNF-α enzyme-linked immunoabsorbent assay (ELISA)

Primary cultured mouse microglia or human-derived THP-1 cells were incubated with indicated concentrations of miRNAs complexed to LyoVec (InvivoGen #LYEC-RNA, San Diego, CA, USA) for indicated durations in 96-well plates (30,000 cells/well). Subsequently, supernatants were collected and stored at − 80 °C. TNF-α amounts were detected via Enzyme-Linked Immunoabsorbent Assay (ELISA; TNF alpha Mouse Uncoated ELISA Kit, Invitrogen, #88–7324-88, Carlsbad, CA, USA) or TNF alpha Human Uncoated ELISA Kit (Invitrogen, #88–7346-88, Carlsbad, CA, USA), according to the manufacturer’s instruction.

### Multiplex immunoassay

Primary cultured microglia (2 × 10^5^ cells/ml) in 96-well plates were incubated with miRNA complexed to LyoVec (InvivoGen #LYEC-RNA, San Diego, CA, USA) at a concentration of 5 μg/ml for indicated durations. Cell-conditioned medium was subsequently collected and analyzed via a customized mouse Procarta Plex Mix and Match 15-plex (eBioscience Inc., San Diego, CA, USA) panel following the manufacturer’s instructions. Briefly, 50 μl magnetic capture beads were plated on a 96-well plate. After washing, 50 μl of cell-conditioned medium were added to each well. The plate was shaken at room temperature for 30 min before being incubated overnight at 4 °C. On the following day, 30 μl of detection antibody mixture were added to each well, and the plate was placed on a shaker at 500 rpm for 30 min. Following washing, the plate was incubated with 50 μl streptavidin/well for 30 min on a shaker, and Reading Buffer was added. Read-out was performed on a Luminex 200 device Bio-Plex Software 4.0 (Bio-Rad Laboratories, Hercules, CA, USA).

### Immunocytochemistry, immunohistochemistry, and apoptosis assay

Immunolabeling was performed as previously described [21]. Anti-neuronal-specific nuclear protein (NeuN; 1:500; Millipore #MAB377, Burlington, MA, USA), anti-Iba1 (1:1000 in vitro, 1:500 for brain sections; cat. #019–19,741, Wako, Neuss, Germany), and anti-TLR7 (1:1000; Novus Biologicals #NBP2–27332, Centennial, CO, USA) were used as primary antibodies in blocking buffer (PBS, 2% Normal Goat Serum, 0.2% TritonX-100). Nuclei were stained with DAPI (1:10,000, Sigma Aldrich, #D9542, St. Louis, MO, USA). Terminal deoxynucleotidyl transferase-mediated biotinylated UTP nick end labeling (TUNEL) apoptosis assay on cell cultures was performed using the In Situ Cell Death Detection kit TMRred (#12156792910 Roche, Basel, Switzerland) following the manufacturer’s instructions. TUNEL apoptosis assay on brain sections was conducted using ApopTag Plus Fluorescein In Situ Apoptosis Detection Kit (Millipore, #S7111, Burlington, USA) following the manufacturer’s instructions. Fluorescence microscopy was performed on an Olympus BX51 microscope (Tokyo, Japan) and on a confocal laser scan Leica TCS SL microscope (Leica Biosystems, Nussloch, Germany) with sequential analysis.

### Phagocytosis bead assay

60,000 microglia were plated on a NuncTM Lab-TekTM II 8 Chamber SlideTM (Thermo Fischer Scientific, #154534PK, Waltham, MA, USA) and incubated with the indicated oligoribonucleotides complexed to LyoVec (InvivoGen #LYEC-RNA, San Diego, CA, USA). After 2 h, microglia were analyzed by phagocytosis bead assay, as described [36]. Briefly, red fluorescent latex beads (size 1 μm; Sigma Aldrich, #L2778, St. Louis, MO, USA) were pre-opsonized in FCS for 1 h at 37 °C. Subsequently, the suspension was diluted at 1:5 in DMEM (Invitrogen #41965062, Carlsbad, CA, USA) and added to microglial cell cultures at 0.01% (v/v). One hour later, microglia were washed 3x with PBS and fixed with 4% paraformaldehyde (PFA). Immunolabeling with Iba1 antibody and subsequent microscopic analysis were performed, as described above. Red signal intensity within Iba1-positive image areas was quantified using FiJi software [37], as described previously [36].

### miRNA tracking in microglial and neuronal endosomes by confocal microscopy

60,000 microglia or 250,000 neurons isolated from C57BL/6 mice were plated per well of a Nunc™ Lab-Tek™ II 8 Chamber Slide™ (Thermo Fischer Scientific, #154534PK, Waltham, MA, USA) and were incubated with 40 μg/ml pHrodo™ Red Dextran (Thermo Fischer Scientific, #P10361, Waltham, MA, USA) for 20 min at 37 °C. Following washing steps, 5 μg/ml fluorescence-labeled miRNAs (Alexa488-miR-298-5p, Alexa488-miR-100-5p or Alexa488-let-7b-5p) complexed to LyoVec (InvivoGen #LYEC-RNA, San Diego, CA, USA) per well were added. After 4 h, cells were washed, stained with DAPI, fixed with 4% PFA, and analyzed by an SP8 confocal laser microscope with sequential analysis (Leica Biosystems, Nussloch, Germany).

### Intrathecal injection of oligoribonucleotides and brain-derived small RNA into mice

Intrathecal injections into mice were performed as described previously [6]. Briefly, 10 μg of synthetic miRNA or control oligonucleotide solved in 40 μl of nuclease-free water were intrathecally injected into 6–8-week-old C57BL/6 or *Tlr7*^*−/−*^ mice. For some experiments, C57BL/6 mice were pre-treated with 1 μg lipopolysaccharide (LPS, 0111:B4, Enzo Life Sciences, Inc., Lörrach, Germany) by intrathecal injection for 16 h before miRNAs were injected, as indicated. Subsequently, mice were sacrificed after 72 h. Following transcardial perfusion with 4% PFA, brains were removed and subsequently treated with sucrose (10–30%) over a course of 3 d for cryoprotection. Slices were immunolabeled with anti-NeuN and anti-Iba1, as well as with DAPI, as described above. In addition, brain sections were analyzed by TUNEL assay, as described above. Cell quantification was performed in a double-blinded fashion and conducted in 6 fields (at 40x magnification) of the right hemisphere of an individual cerebral cortex.

For experiments involving intrathecal injection of brain-derived small RNA, total RNA from adult C57BL/6 cerebral cortices was extracted using Trizol, followed by small RNA enrichment using the *mir*Vana miRNA Isolation kit (Thermo Fisher Scientific, Waltham, MA, USA), as described above. Six to 8 weeks-old male C57BL/6 mice were intrathecally injected with 125 pmol of miRCURY locked-nucleic acid (LNA) Power Inhibitor specific for mmu-miR-298-5p (GGCAGAGGAGGGCUGUUCUUCCC; #339131, Qiagen, Hilden, Germany), or miRCURY LNA miRNA Control Inhibitor (TAACACGTCTATACGCCCA; #339136, Qiagen), as described previously [6, 8]. After 16 h, mice were injected for a second time with 10 μg of brain-derived small RNA. Sham-operated mice received 2 consequent solvent (H_2_O) injections. After a further 3 days, mice were sacrificed, and brains were analyzed by immunohistochemistry using a NeuN antibody, as described above.

### Microscale thermophoresis (MST)

Purified polyhistidine-tagged human TLR8 protein (LSBio, #LS-G23167, Seattle, WA, USA) was delivered in Tris/HCl buffer with 50% glycerol. Receptor labeling was performed following the manufacturer’s protocol (RED-tris-NTA dye, #MO-L008, NanoTemper Technologies GmbH, Munich, Germany). 2 μM TLR8 was diluted at 1:10 in MST buffer (50 mM Tris/HCl pH 7.4, 150 mM NaCl, 10 mM MgCl_2_, 0.05% polysorbate (Tween) 20, 0.06% n-Dodecyl-beta-Maltoside) to a final concentration of 200 nM. RED-tris-NTA was dissolved in 1x PBS-T buffer to a concentration of 100 nM, mixed 1:1 with TLR8 protein, and incubated on ice for 30 min. MST measurements were performed in the cold room under the controlled ambient temperature of 10 °C. To prevent nucleotide oligomerization, each oligoribonucleotide was incubated for 5 min at 80 °C and placed on ice until experimental start. RNA was titrated with a serial dilution of RNA with H_2_O. For each single measurement 5 μl of RNA were mixed with 5 μl RED-tris-NTA-labeled TLR8 and incubated on ice for 5 min, shortly before the sample was loaded to standard glass capillaries. Instrumentation and experimental settings: Monolith NT.115 MST_power_ = medium; LED_power_ = 100%. RED-tris-NTA labeling kit and capillaries were purchased from NanoTemper Technologies GmbH (#MO-K022, Munich, Germany). Data analysis was performed with NanoTemper MO Affinity Analysis V2.3 software and visualized using SigmaPlot V13.0.

### Statistical analysis

Data are expressed as mean ± SD. Absolute values or normalized data to control conditions are depicted, as indicated in the respective figure. Statistical differences over all groups were analyzed with one-way ANOVA with Holm-Sidak’s multiple comparison tests. Statistical differences between two specific groups were analyzed with the parametric two-tailed Student’s *t*-test. Statistics were performed using GraphPad Prism 7.0 and 8.0 (GraphPad Software, LLC).

## Results

### Identification of miRNAs released from apoptotic cortical neurons by miRNA microarray

**Fig. 1:**
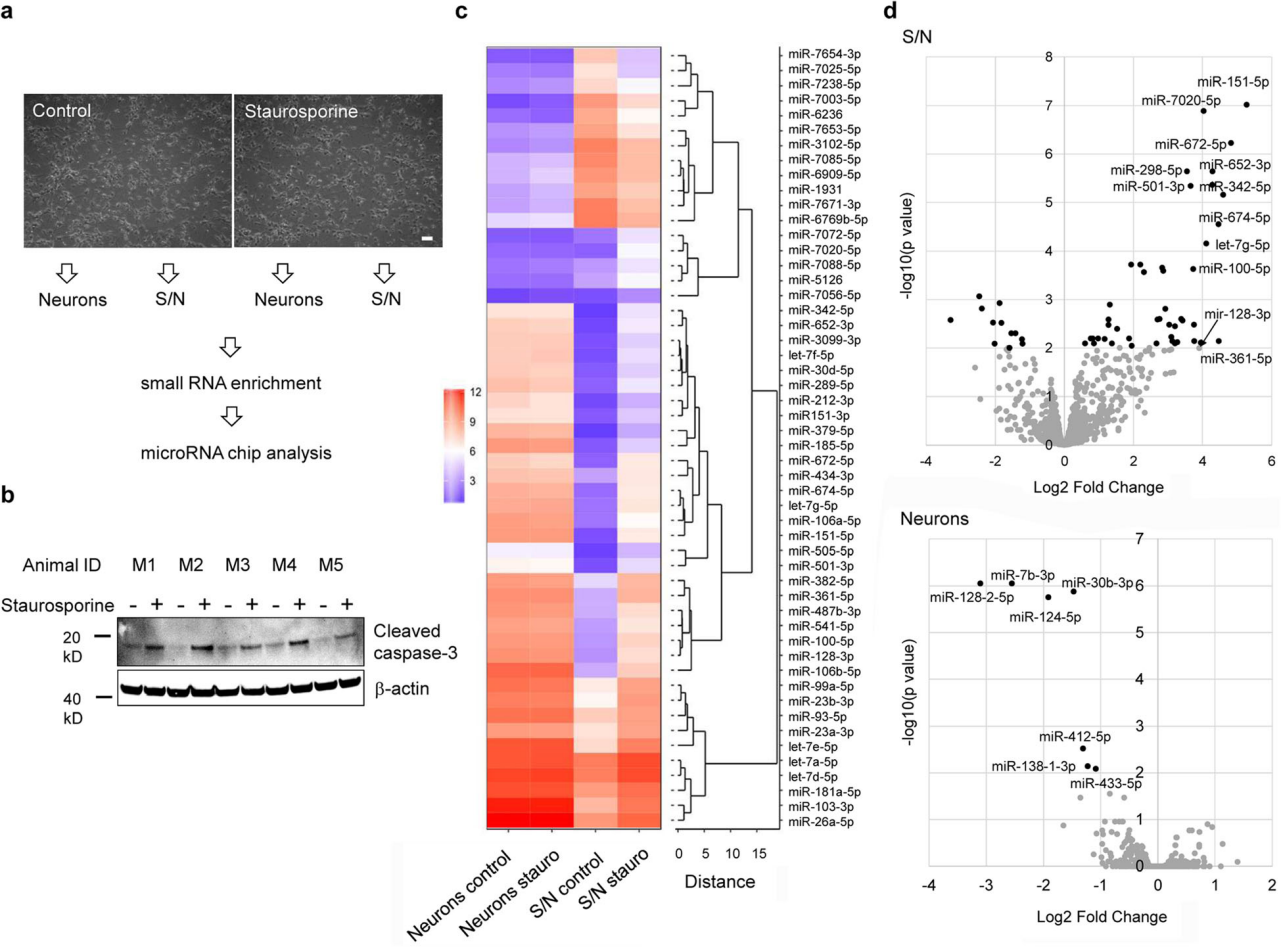
Identification of miRNAs expressed in apoptotic cortical neurons and present in corresponding supernatant by miRNA microarray. (**a**) Representative images of cortical neurons isolated from C57BL/6 mice incubated with either 1 μM staurosporine or DMSO (0.1%) as solvent control for 8 h. Scale bar, 20 μm. Neurons and corresponding supernatant (S/N) were collected separately for small RNA enrichment and subsequent miRNA GeneChip analysis, as indicated by arrows. (**b**) Immunoblot depicting cleaved caspase-3 in C57BL/6 cortical neurons derived from 5 individual cell cultures after staurosporine (+) or DMSO (−) treatment. β-actin served as loading control. (**c**) Heat map of significantly differentially expressed miRNAs in apoptotic cortical neurons and corresponding S/N (*P* < 0.01, Log2 Fold Change > 1) described above. Color scale corresponds to the robust multi-array average-processed miRNA expression values after averaging over replicates. Unstimulated neurons (control, *n* = 5), staurosporine-treated neurons (*n* = 5), control S/N (*n* = 5), apoptotic neuron S/N (*n* = 4). miRNA IDs are depicted for significantly deregulated miRNAs in the S/N of apoptotic cortical neurons. (**d**) Volcano plots with significantly differentially expressed miRNA species indicated by black filling in apoptotic neurons and their conditioned S/N (*P* < 0.01, Log2 Fold Change > 1)

As miRNA expression is altered in brains of patients with neurodegenerative diseases, and some extracellular miRNAs, such as *let-7b,* were shown to induce TLR signaling [6, 24], we aimed to systematically identify miRNAs that act as novel TLR ligands in CNS injury. To this end, primary cortical neurons isolated from C57BL/6 mice were first exposed to staurosporine, a potent, non-selective kinase inhibitor, which is widely used to model neuronal apoptosis, for 8 h. While neurons remained morphologically intact within this time period (Fig. 1a), induction of apoptosis through activation of neuronal caspase-3 signaling was verified by western blot (Fig. 1b). Next, neurons and corresponding supernatant (S/N) were separately harvested and processed for miRNA detection (Fig. 1a), as previously described [24]. After enrichment and isolation of the small RNA fractions from neurons and their corresponding S/N, they were analyzed by the latest version of GeneChip miRNA 4.0 array technology (Fig. 1c). Quality control after hybridization confirmed an average distribution of the normalized signal intensities of expression values on the analyzed gene chips, for the different experimental conditions (Additional file 1a). Principal component analysis revealed that the S/N control and S/N staurosporine groups formed clusters. In case of control neurons and staurosporine-treated neurons no clear group separation was observed (Additional file 1b). Setting a cut-off at *P* < 0.01 (absolute Log2 Fold Change > 1), 39 miRNAs were detected as significantly more abundant and 12 miRNAs as significantly less abundant in the S/N of apoptotic cortical neurons compared to the S/N of untreated cortical neurons (control, Fig. 1d). In addition, expression of seven miRNAs was reduced in apoptotic cortical neurons, when applying the cut-off criteria described above (Fig. 1d; Additional Table 1).

The accumulation of miRNAs in S/N of apoptotic neurons might be due to nonspecific release of the entire miRNA content physiologically present in untreated neurons upon cell death induction. To test for such a nonspecific release, we correlated the average miRNA expression values determined under neuron control conditions with the Log2 Fold Change values for the 39 miRNAs significantly enriched in the S/N of apoptotic cortical neurons by calculating the Pearson correlation coefficient (Additional file 2). No correlation between the abundance of significantly enriched miRNAs selected using the cut-off criteria (*P* < 0.01, Log2 Fold Change > 1; 39 miRNAs) described above in S/N of apoptotic neurons and their abundance in untreated control neurons was detected (Additional file 2a, Additional Table 1). Even after relaxing the cut-off criteria to a lower significance threshold (*P* < 0.05), thereby including a total number of 88 miRNAs enriched in apoptotic neuron S/N in our analysis, no correlation between these 88 miRNAs and miRNAs detected in control neurons was observed (Additional file 2b). These findings indicate a specific, regulated release of miRNAs from apoptotic cortical neurons rather than a general, nonspecific discharge of the whole neuronal miRNA content into the extracellular space.

Among the 39 miRNAs significantly enriched in the conditioned media of apoptotic cortical neurons, five *let-7* miRNA family members were detected, namely *let-7a*, *let-7d*, *let-7e*, *let-7f*, and *let-7 g* (Fig. 1c, d; Additional Table 1). All of these oligoribonucleotides directly activate TLR7 in microglia [38]. In addition, miRNAs miR-128-2-5p, miR-124-5p, miR-30-3p, and miR-7b-3p were significantly less abundant in apoptotic cortical neurons compared to untreated control neurons (Fig. 1d), suggesting that these miRNA species may have also been released as a consequence of apoptosis induction. We assumed the 39 miRNAs enriched in S/N of apoptotic cortical neurons to be the most potent candidates to act as TLR ligands mediating cell-to-cell communication in the brain. To determine the biological processes, in which the 39 candidate miRNAs are involved in, Gene Ontology (GO) analysis was applied using in-house software, which identifies processes regulated by miRNA-RNA target interactions by integrating information from different databases. The tested candidate miRNAs were linked to GO biological processes terms such as regulation of cell communication, programed cell death, and synaptic signaling (Additional file 3), highlighting their potential to act as extracellular signaling molecules.

### miRNAs released from apoptotic cortical neurons activate human TLR7 and/or TLR8

Some ssRNAs, including miRNAs, activate TLR7 and TLR8 in a sequence-specific manner [6, 8, 14]. As TLR activation can in principle contribute to human CNS disorders, we sought to determine whether the miRNAs released from apoptotic cortical neurons identified above directly activate human TLR7/8. To this end, out of the 39 miRNAs enriched in the S/N of apoptotic neurons detected by miRNA microarray (see above), one top list of ten miRNAs was selected based on absolute logarithmic Fold Change values. A second top list of ten miRNAs was selected based on their levels of significance (see Fig. 1c, d). Eight miRNAs appeared on both lists, while two miRNAs each appeared on one list, respectively. Thus, a final list of 12 candidate miRNAs emerged (Table 1), which were tested for their capacity to activate human TLR7 and/or human TLR8 in the following.

**Fig. 2:**
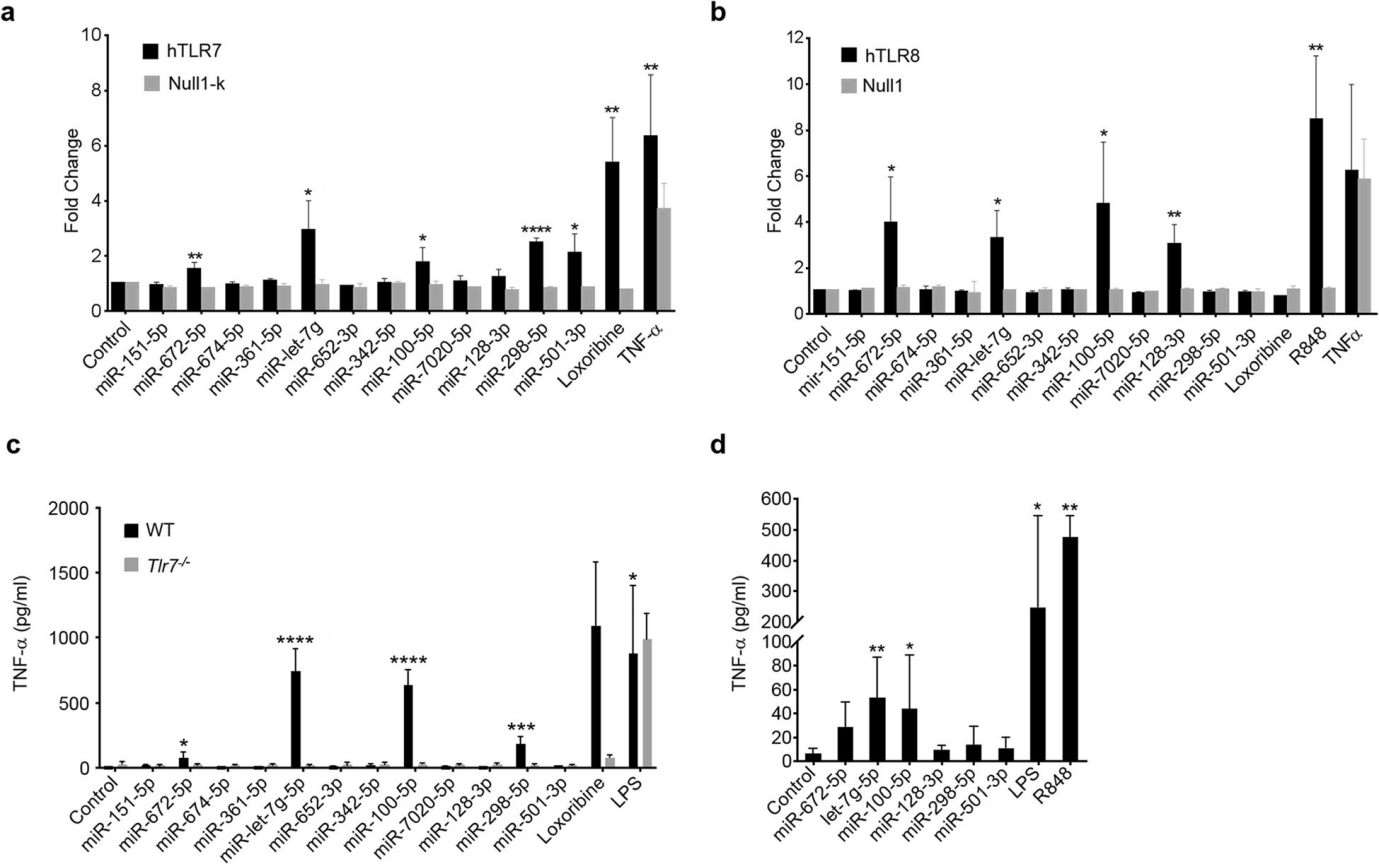
Extracellular miRNAs activate human TLR7 and TLR8 expressed in HEK TLR reporter cells, as well as murine microglia and human monocytes, depending on their sequence. HEK-Blue cells co-expressing human TLR7 **a** or human TLR8 **b** and an NF-κB/AP1-inducible secreted embryonic alkaline phosphatase (SEAP) reporter gene (**a**, **b**) were incubated with 20 μg/ml of indicated miRNAs (for miRNA concentrations given in [nm] please refer to Additional Table 2), loxoribine (**a**, **b**, 1 mM, TLR7 agonist), R848 (**b**, 100 ng/ml, TLR7/8 agonist), or TNF-α (**a**, **b**, 100 ng/ml, SEAP induction) for 24 h. Unstimulated HEK-Blue TLR-expressing cells and HEK-Blue Null1 or Null1-k cells served as negative control. Data are expressed as fold change of optical density of the SEAP protein normalized to unstimulated control. Data are represented as mean ± SD (*n* = 3). *P < 0.05; **P < 0.01; ****P < 0.0001 compared to the corresponding Null1 or Null1-k control, two-tailed Student’s *t*-test. (**c**) Microglia from C57BL/6 (wild-type, WT, *n* = 4) or *Tlr7*^*−/−*^ (*n* = 3) mice were incubated with 5 μg/ml of indicated miRNAs for 24 h followed by TNF-α ELISA. Unstimulated cells served as negative control. LPS (100 ng/ml) and loxoribine (1 mM) served as positive control. Data are expressed as mean ± SD. *P < 0.05; ***P < 0.001; ****P < 0.0001 compared to control, Student’s *t*-test. (**d**) Macrophages differentiated from THP-1 cells were incubated with 5 μg/ml of indicated miRNAs for 24 h. Unstimulated cells served as negative control. LPS (100 ng/ml) and R848 (100 ng/ml) served as positive control. TNF-α amounts in supernatants were subsequently quantified by ELISA. All results are shown as mean ± SD with *n* = 6. *P < 0.05; **P < 0.01 compared to control, one-way ANOVA with Holm-Sidak’s multiple comparison test

To test for human TLR7 and/or TLR8 activation by the candidate miRNAs determined above, we utilized a HEK-Blue Secreted Embryonic Alkaline Phosphatase (SEAP) reporter cell assay overexpressing human TLR7 or human TLR8, as well as Null1-k or Null1 reporter cells serving as the negative control. The SEAP gene was inserted directly after the NF-κB/AP-1 promoter, ensuring that TLR activation leads to SEAP secretion, which in turn could be detected via colorimetric analysis. Six of the 12 miRNAs selected for testing human TLR7/TLR8 activation induced significant activation of at least one of the receptors (Fig. 2a, b). While let-7g-5p, miR-100-5p, and miR-672-5p consistently activated both human TLR7 and TLR8 (Fig. 2a, b), we observed exclusive activation of human TLR7 by miR-298-5p and miR-501-3p (Fig. 2a). In contrast, miR-128-3p-induced receptor activation was restricted to human TLR8 (Fig. 2b). Neither TLR7 nor TLR8 were activated by miR-151-5p, miR-674-5p, miR-361-5p, miR-652-3p, miR-342-5p, or miR-7020-5p (Fig. 2a, b). Notably, the receptor-activating miRNAs miR-672-5p, let-7g-5p, miR-100-5p (TLR7/8), and miR-298-5p (TLR7) all contain GU-rich and/or AU-rich sequence motifs of at least 4-nucleotide length (Table 1, sequence motifs highlighted in bold), which have been previously described to induce TLR7 and TLR8 signaling in human peripheral blood mononuclear cells [11]. However, although miR-361-5p and miR-652-3p possess previously described TLR activation motifs (Table 1), they did not induce TLR7/8 signaling in HEK TLR reporter cells (Fig. 2a, b). In contrast, miR-501-3p, which does not harbor any of the previously described TLR-activating motifs, activated human TLR7 (Table 1, Fig. 2a, b). These findings suggest that the presence of a known TLR-activating motif per se is not sufficient for activation of TLR7/8 by a specific miRNA.

In summary, six out of 12 tested miRNAs released from mouse apoptotic cortical neurons activated human TLR7 and/or human TLR8.

### Novel miRNA-based TLR7/8 activators including miR-100-5p and miR-298-5p induce cytokine and chemokine release from mouse microglia and human-derived macrophages

Microglia are crucial players in neuroinflammation and CNS damage. Both human and mouse microglia express a broad range of TLRs, including TLR7 and TLR8 [39, 40]. Primary murine microglia represent a well-established model to test microglial function in the human brain [41], albeit expressing only functional TLR7, while lacking TLR8-mediated signaling [9]. To assess the functional consequences of TLR activation in the CNS by the 12 miRNAs released from apoptotic cortical neurons selected above (see Table 1), we first tested the miRNAs’ ability to activate microglia. To this end, C57BL/6 microglia were exposed to the respective synthetic oligoribonucleotide, as indicated, for 24 h, and microglial S/N were subsequently analyzed for their TNF-α content via ELISA. miR-672-5p, let-7g-5p, miR-100-5p, and miR-298-5p induced TNF-α release from microglia (Fig. 2c). This inflammatory response induced by the respective oligoribonucleotide required TLR7, as cytokine release was completely abolished in *Tlr7*^*−/−*^ microglia (Fig. 2c). TNF-α release induced by the miRNAs named above was time- (Additional file 4a) and dose-dependent (Additional file 4b). The results on the effects on mouse microglia exposed to miR-100-5p, miR-298-5p, miR-672-5p, and let-7 g-5p through TLR7 were in line with their ability to activate human TLR7 expressed in HEK-Blue TLR reporter cells (see Fig. 2a). In contrast, miR-151-5p, miR-674-5p, miR-361-5p, miR-652-3p, miR-342-5p, miR-7020-5p, miR-128-3p, and miR-501-3p did not induce TNF-α release from mouse microglia (Fig. 2c).

Next, we investigated the potential of the six miRNAs that had elicited a response in human TLR7/8 expressed in HEK-Blue TLR reporter cells, namely miR-100-5p, miR-672-5p, miR-298-5p, let-7 g-5p, miR-128-3p, and miR-501-3p (see Fig. 2a, b), to activate human immune cells. To this end, human monocyte-derived THP-1 cells differentiated into macrophages were used, as they express both TLR7 and TLR8 [42, 43]. THP-1 cells were incubated with the above-mentioned miRNAs for 24 h, and TNF-α amounts in the S/N were assessed by ELISA. In accordance with our findings on activation of human TLR7 and TLR8 expressed in the HEK TLR reporter cells, let-7 g-5p and miR-100-5p induced TNF-α release from THP-1-differentiated macrophages (Fig. 2d). Although not reaching statistical significance, miR-672-5p exposure also resulted in increased TNF-α release compared to control condition. In contrast, miR-298-5p and miR-501-3p, which exclusively had activated human TLR7 expressed in the HEK TLR reporter cells (see Fig. 2a), were unable to induce such a response in THP-1 cells (Fig. 2d). These findings and the inability of miR-128-3p to induce TNF-α release from THP-1 cells (Fig. 2d), despite its ability to activate human TLR8 in HEK TLR reporter cells (see Fig. 2b), point to cell type-specific differences in miRNA-mediated TLR7/8 activation.

**Fig. 3:**
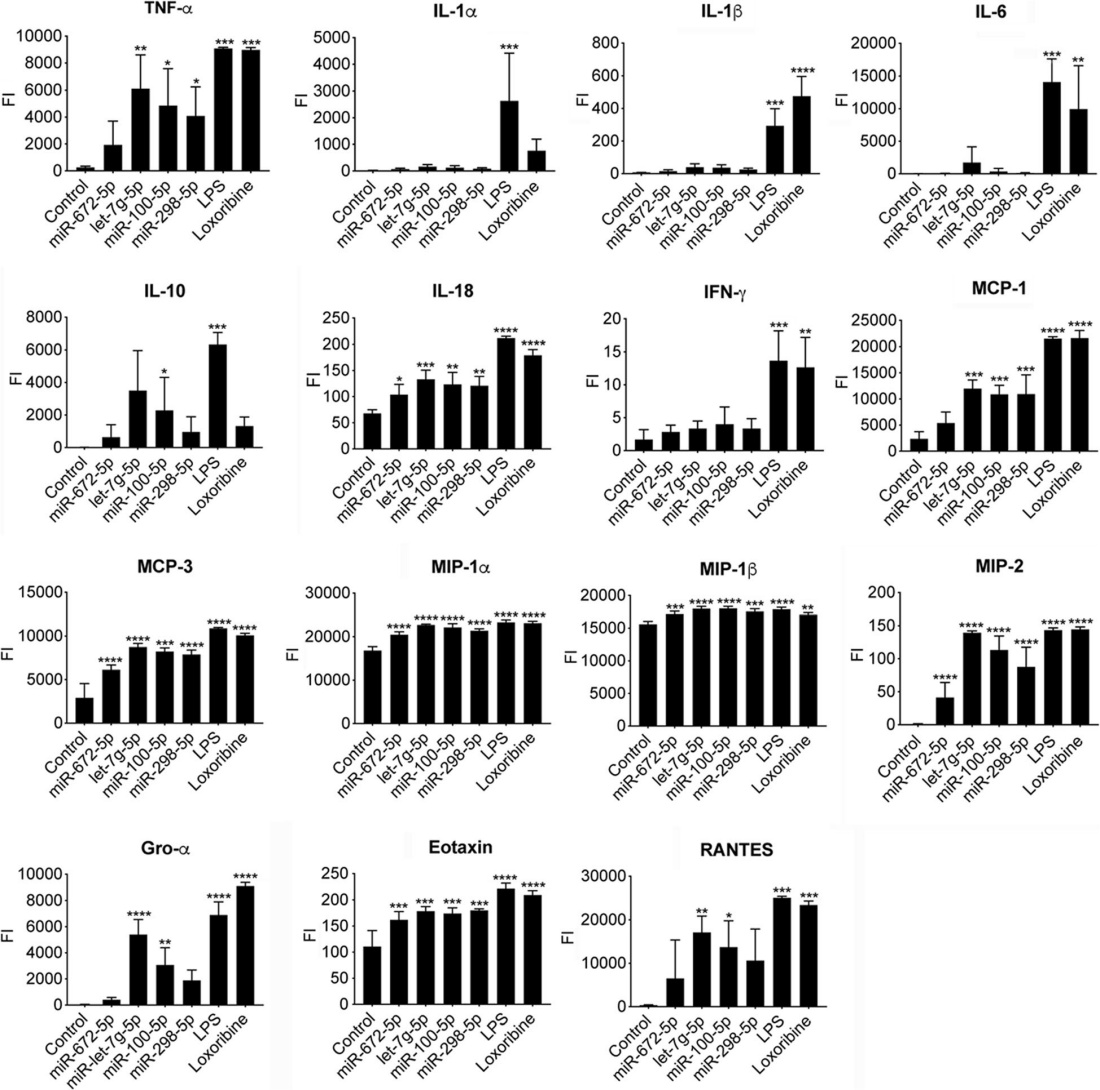
MiRNAs miR-672-5p, let-7 g-5p, miR-100-5p, and miR-298-5p induce cytokine and chemokine release from microglia. Microglia isolated from C57BL/6 mice were incubated with 5 μg/ml of candidate miRNA, as indicated, for either 24 h (Gro-α, IFN-γ, IL-6, MIP-2) or 36 h (other cytokines/chemokines). The TLR7 agonist loxoribine (1 mM) and the TLR4 agonist LPS (100 ng/ml) served as positive control. Unstimulated cells were used as negative control. The release of depicted inflammatory mediators was subsequently analyzed by multiplex immune assay. Data are shown as fluorescent intensity (FI) values expressed as a. u., arbitrary units, mean ± SD (*n* = 3). *P < 0.05; **P < 0.01; ***P < 0.001; ****P < 0.0001 compared to negative control, one-way ANOVA with Holm-Sidak’s multiple comparison test

We sought to determine the specific patterns of inflammatory molecules released from microglia in response to those miRNAs that had elicited TNF-α production (see Fig. 2c and Additional file 4). Using a multiplex immunoassay capable of detecting 15 different cytokines and chemokines previously linked to microglial activation and neuroinflammation [38], we found that miR-672-5p, let-7 g-5p, miR-100-5p, and miR-298-5p induced a specific neuroinflammatory pattern (Fig. 3). All tested miRNAs triggered the release of IL-18, MCP-3, MIP-1α, MIP-1β, MIP-2, and Eotaxin from microglia. In contrast, IL-1α, IL-1β, IL-6, and IFNγ were not induced by any of the miRNAs. TNF-α and MCP-1 were significantly released upon exposure to miR-100-5p, miR-298-5p, and let-7 g-5p. Although not reaching statistical significance, incubation of microglia with miR-672-5p also led to an increased TNF-α and MCP-1 production compared to control. Gro-α and RANTES release was restricted to microglia exposed to miR-100-5p and let-7 g-5p. While only miR-100-5p induced a statistically significant IL-10 response in microglia, incubation with miR-298-5p, let-7 g-5p, and miR-672-5p showed a tendency towards increased microglial IL-10 release (Fig. 3).

In summary, miR-100-5p, miR-298-5p, miR-672-5p, and let-7g-5p induced the release of specific inflammatory mediators from mouse microglia. miR-100-5p, let-7 g-5p, and to a lesser extent miR-672-5p, activated human macrophages. These results highlight the potential of the miRNAs identified as novel TLR7/8 activators to contribute to neuroinflammation in mouse and human.

### TLR7/8 activation through extracellular miRNAs requires novel sequence features, but no specific structure formation of mature miRNA

TLR7 expressed in murine microglia can be activated by the sequence motif UUGU [6, 38]. However, further GU-rich 4-nucleotide motifs have been shown to activate TLR7 and TLR8 in human immune cells [11]. As described above, six out of 12 of our candidate miRNAs released from apoptotic cortical neurons induced human TLR7/8 activation (see Fig. 2a, b). Among those miRNAs, miR-100-5p, miR-298-5p, miR-672-5p, and let-7 g-5p also activated murine microglia, which express only TLR7. Based on these findings, we analyzed whether the above-mentioned miRNAs contain the UUGU motif. Among human TLR7/8- and mouse microglia-activating miRNAs, only let-7 g-5p and miR-100-5p harbor this exact minimal motif (Table 1). This supports previous suggestions that further nucleotide subsequences/motifs are important for TLR7/8 activation [38]. Not only pre-miRNA sequences, but also the shorter, mature miRNA can form secondary structures [44]. We therefore aimed at systematically analyzing whether miRNA structure is required for TLR7/8 activation, or has any impact on receptor activation. To this end, we computed secondary structure predictions via the RNAfold webserver [45], which identifies the most stable RNA secondary structure and provides a respective minimal free energy estimate for each calculated miRNA (Table 1). Lower energy values reflect more stable structures, while especially energies below − 5 kcal/mol are considered thermodynamically stable. Out of the 12 miRNAs released from apoptotic cortical neurons and selected for follow-up experiments on TLR activation performed within this study, only miR-298-5p falls below this threshold (Table 1). Thus, although differentiation between activating and non-activating miRNAs based on minimum free energy values is not feasible, our findings indicate that miRNAs do not necessarily form a distinct secondary structure, e.g. in order to present the interacting motif to the receptor or to activate the respective TLR.

### Extracellular miR-100-5p and miR-298-5p enter microglia, localize to their endosomes, and directly bind to TLR8

In immune cells, TLR7 and TLR8 localize to endosomes [2]. Therefore, miRNAs must enter the endosomal pathway before they can directly activate TLR7/8. Out of the six miRNAs identified as novel TLR7/8 activators, we selected miR-100-5p and miR-298-5p to pursue further studies with respect to their functional relevance in neuroinflammation and CNS injury for the following reasons: i) both miRNAs activate human TLR7 and/or human TLR8, ii) both miRNAs induce cytokine release from mouse microglia, thereby triggering an inflammatory response, iii) miR-100-5p activates not only mouse microglia but also human-derived macrophages, and iv) both miRNAs were previously linked to neurodegenerative diseases, including AD [46–50]. To further investigate the miRNA candidates’ potential to activate microglia (see Fig. 3 and Additional file 5a), we tested low doses of miR-100-5p and miR-298-5p. Both miRNAs induced TNF-α release at doses as low as 0.1 μg/ml (13.48 nM, miR-100-5p; 12.77 nM, miR-298-5p; Additional file 5b). In comparison, HEK TLR reporter cells expressing hTLR7 responded to miRNA doses as low as 1 μg/ml (127.75 nM, miR-298-5p) and 5 μg/ml (673.99 nM, miR-100-5p), as assessed by NF-*k*B induction. HEK reporter cells expressing hTLR8 were significantly activated by doses starting from 0.1 μg/ml (13.48 nM, miR-100-5p; Additional file 5c).

Having observed that the miRNA candidates serving as TLR7/8 ligands induced the release of cytokines from microglia, we wondered whether miR-100-5p and miR-298-5p modulate further microglial functions, such as phagocytosis. Thus, microglia were exposed to miR-100-5p and miR-298-5p for 2 h, and subsequently were incubated with fluorescent beads for another 1 h. LPS served as positive control. Bead quantification by fluorescence microscopy revealed that exposure to both miRNAs significantly increased the phagocytic activity of microglia (Additional file 6).

**Fig. 4:**
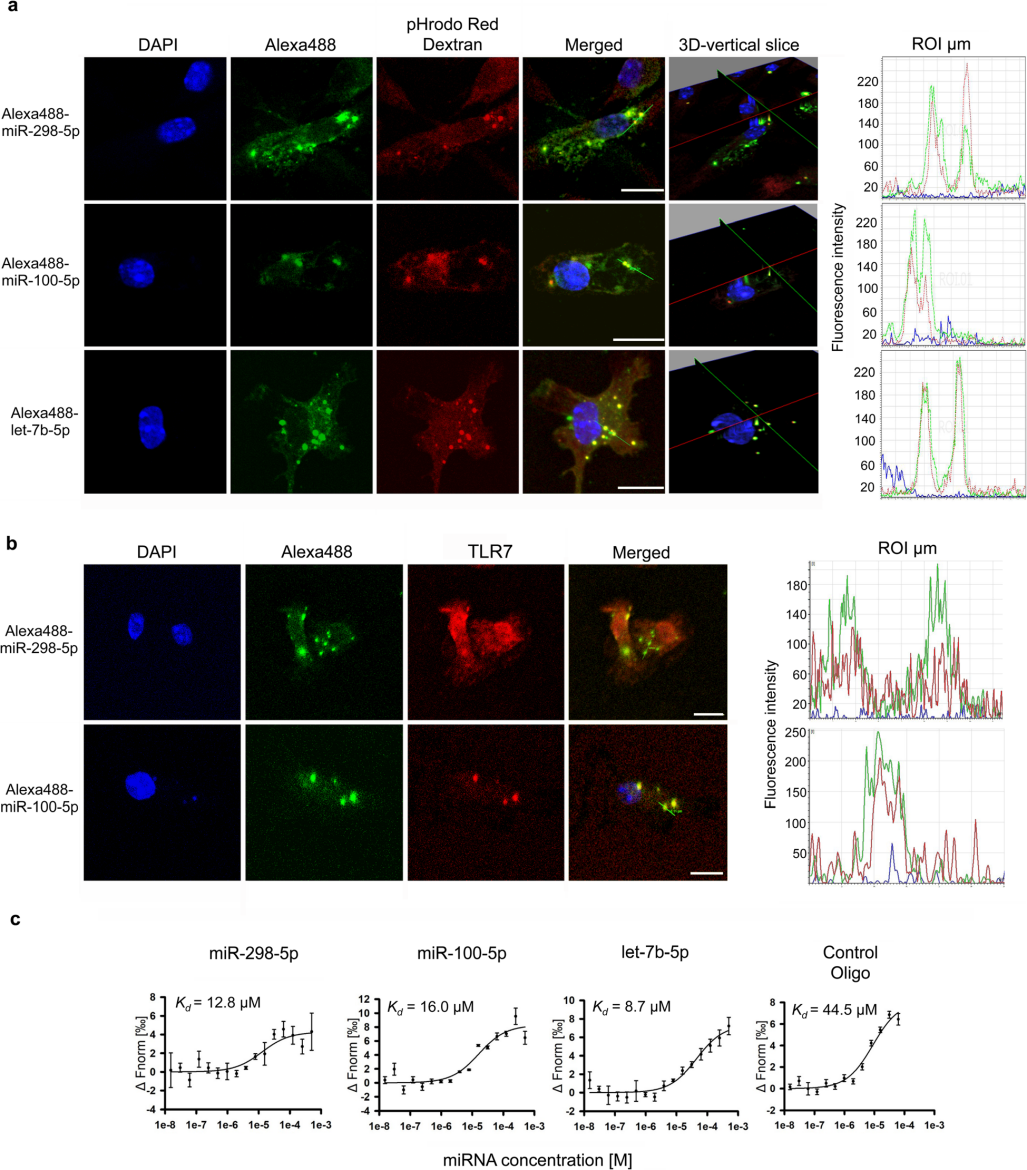
miR-298-5p and miR-100-5p enter microglia, co-localize to their endosomes, and bind directly to human TLR8. **a** C57BL/6 microglia were incubated with 40 μg/ml pHrodo Red Dextran serving as endosomal marker for 20 min. Subsequently, microglia were exposed to 5 μg/ml of Alexa488-labeled miR-298-5p, Alexa488-labeled miR-100-5p, or Alexa488-labeled let-7b-5p for 4 h. Cells were then fixed and stained with DAPI. **b** In parallel, microglia exposed to Alexa488-labeled miR-298-5p or Alexa488-labeled miR-100-5p, as described above, were fixed and immunolabeled with TLR7 antibody. (**a**, **b**) Cells were analyzed by confocal microscopy with sequential analysis. Representative images of microglia incubated with the indicated fluorescent miRNAs (488 nm, green), stained with DAPI (405 nm, blue), and pHrodo Red Dextran or TLR7 (552 nm, red) are depicted. Green lines indicate region of interest (ROI); scale bar, 10 μm. Representative 3D-vertical slice images of microglia show co-localization of miRNAs and endosomes through different cellular levels (left panel). Diagrams depict fluorescence intensities of the marked ROI in microglia for the sequential analysis used (DAPI: blue line; pHrodo Red Dextran/TLR7: red line; fluorescent miRNA: green line, right panel). (**c**) Binding affinity measurements of purified polyhistidine-tagged human TLR8 protein and synthetic miRNAs, as indicated, using microscale thermophoresis (MST). TLR8-RNA interaction was monitored by titrating indicated miRNAs from 500 μM to 30 nM against 50 nM RED-tris-NTA-labeled hTLR8 protein and measured with the NanoTemper Monolith NT.115 MST device. *K*_*d*_ values were calculated from dose response curves, which were generated from titration experiments. Data are expressed as mean ± SD, *n* = 4

Next, we sought to visualize the interaction between extracellularly delivered miR-100-5p and miR-298-5p and microglia. To this end, we exposed mouse microglia to these two miRNAs tagged with the fluorochrome Alexa488 on their 5′ end, respectively, for 4 h. Alexa488-labeled let-7b-5p*,* previously reported as a direct TLR7 activator [7], was included as a comparative condition in this experimental set-up (Fig. 4a). Confocal microscopy generating 2D- and 3D-vertical slice images revealed that both candidate miRNAs, but also let-7b-5p, indeed entered microglial cells within the defined time period (Fig. 4a). Furthermore, all three fluorescently tagged miRNAs localized to the endosomal compartment marked with pHrodo Red Dextran [51], as confirmed by fluorescence intensity spectrum analysis (Fig. 4a). Immunolabeling of microglia using a TLR7 antibody demonstrated co-localization of miR-100-5p and miR-298-5p with TLR7 (Fig. 4b).

To determine the binding affinities between TLRs and miR-100-5p and miR-298-5p, we performed microscale thermophoresis (MST). In detail, a purified polyhistidine-tagged (His-Tag) TLR8 protein fragment containing leucine-rich repeats harboring known ligand recognition sites [8] was fluorescently labeled with RED-tris-nitrilotriacetic acid (RED-tris-NTA), which binds to the His-Tag with high affinity. Subsequently, TLR-miRNA interaction was monitored by titrating synthetic unlabeled miR-298-5p and miR-100-5p using indicated concentrations (Fig. 4c). let-7b-5p served as positive control for TLR binding. We assessed a *K*_*d*_ = 12.8 μM for miR-298-5p, a *K*_*d*_ = 16.0 μM for miR-100-5p, and a *K*_*d*_ = 8.7 μM for let-7b-5p. Compared to these findings, the negative control oligonucleotide harboring a mutated let-7b-5p sequence, which does not activate TLR7/8 [6, 8], bound with lower affinity (*K*_*d*_ = 44.5 μM), as expected (Fig. 4c). MST for let-7g-5p, which efficiently activated TLR7/8 expressed in HEK cells and microglia (see Figs. 2 and 3), yielded a lower *K*_*d*_ of 30.2 μM, indicating that binding of a given miRNA to the receptor and receptor activation are not necessarily interdependent (Additional file 7a). Specificity of the MST measurement was validated using miR-298-5p, which was titrated in the presence of a control (His-Tag labeling kit) RED-tris-NTA-labeled peptide. The affinity of miR-298-5p to the control peptide resulted in a *K*_*d*_ value of 320 μM (Additional file 7b).

Taken together, extracellular miR-100-5p, miR-298-5p, and let-7b-5p entered microglia and located to their endosomes. Furthermore, these miRNAs, as well as let-7 g-5p, directly bound to human TLR8, thereby serving as its immediate ligands.

### miR-100-5p and miR-298-5p induce neuronal injury in vitro

**Fig. 5:**
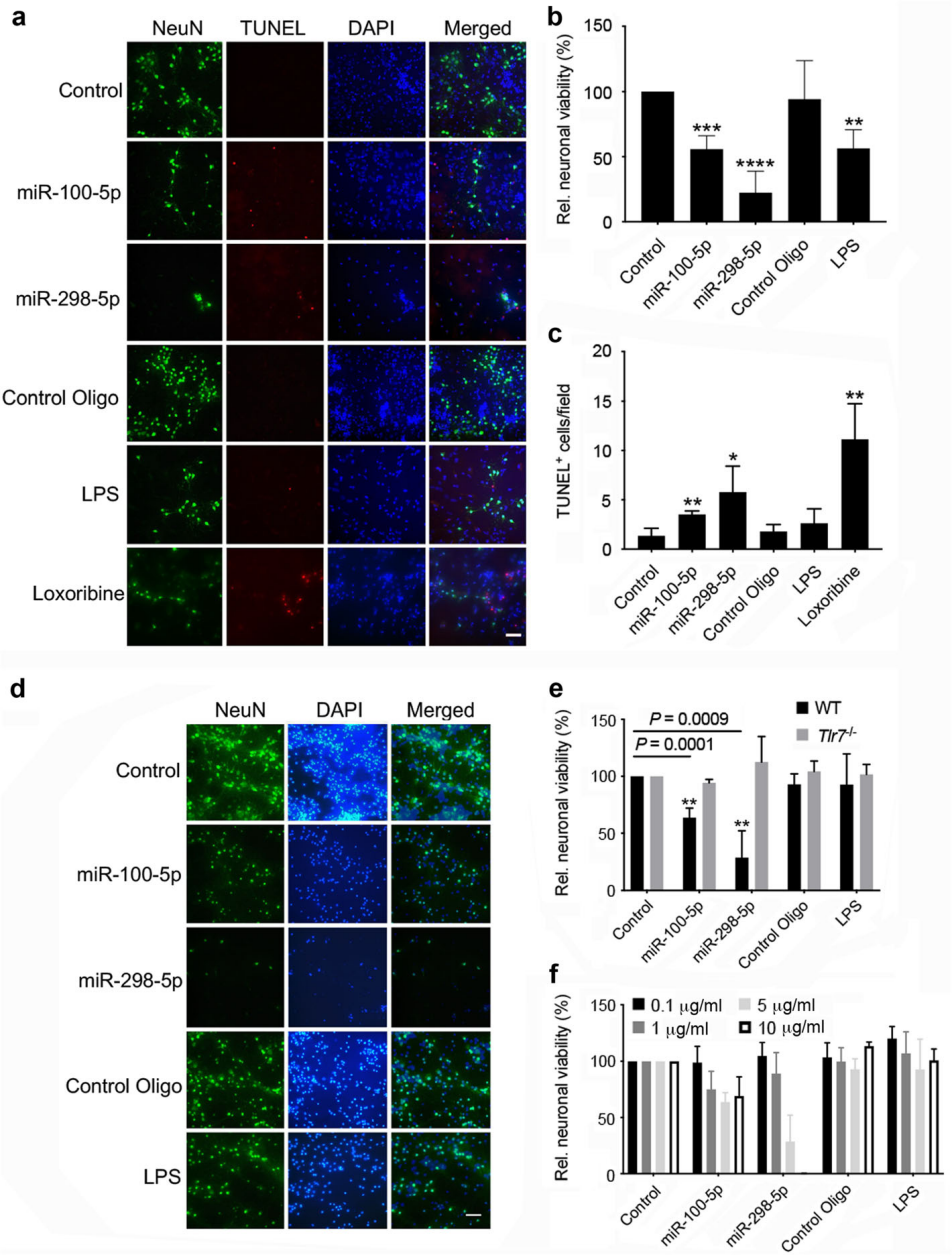
miR-100-5p and miR-298-5p induce neuronal injury in vitro. **a** Representative images of C57BL/6 (wild-type, WT) cortical neurons co-cultured with WT microglia. Cells were incubated with 5 μg/ml of indicated miRNAs for 5 d. Cell cultures were subsequently immunostained with NeuN antibody (green) and stained with TUNEL apoptosis assay (red) and DAPI (blue). LPS (100 ng/ml) and loxoribine (1 mM) served as positive control. Mutant control oligonucleotide and unstimulated cells were used as negative control. Scale bar, 20 μM. **b**, **c** Quantification of NeuN- and TUNEL-positive neurons in co-cultures containing neurons and microglia. Data are expressed as mean ± SD, *n* = 4. *P < 0.05; **P < 0.01; ***P < 0.001, ****P < 0.0001 compared to control, two-tailed Student’s *t*-test. **d** Representative images of enriched WT cortical neurons, which were incubated with 5 μg/ml of indicated miRNAs for 5 d. Cell cultures were subsequently immunostained with NeuN antibody (green) and stained with DAPI (blue). LPS (100 ng/ml) was used to test for potential relevant contamination with microglia. Mutant control oligonucleotide and unstimulated cells were used as negative control. Scale bar, 20 μM. **e** Both WT and *Tlr7*^*−/−*^ neurons were incubated with 5 μg/ml of indicated miRNAs for 5 d. LPS (100 ng/ml) was used to test for potential relevant contamination of the enriched neuron cultures with microglia. Mutant control oligonucleotide and unstimulated cells were used as negative control. Subsequently, NeuN-positive cells in WT and *Tlr7*^*−/−*^ cortical neuron cultures were quantified. Data are depicted as relative neuronal viability determined in cell cultures treated with the indicated miRNA compared to control. Results are shown as mean ± SD (*n* = 4 for WT, *n* = 3 for *Tlr7*^*−/−*^ neurons). **P < 0.01 WT vs. *Tlr7*^*−/−*^*. P* values for relevant groups as determined using the two-tailed Student’s *t*-test are shown. **f** Enriched WT cortical neurons were incubated with indicated concentrations of synthetic miRNAs for 5 d. Cultures were immunostained as described above, and NeuN-positive cells were quantified. LPS was used to test for potential relevant contamination with microglia. Mutant control oligonucleotide and unstimulated cells were used as negative control. Data are depicted as relative neuronal viability determined in cell cultures treated with the indicated miRNA. Results are shown as mean ± SD, *n* = 4

We have shown in previous work that various TLRs including TLR4 and TLR7 expressed in microglia can induce neuronal injury [6, 21, 52]. Thus, we next investigated the functional impact of the miRNAs identified as novel TLR7/8 ligands and activators of mouse microglia, as described above (see also Fig. 2c), in a mouse model of neuronal injury in vitro. We exposed co-cultures containing cortical neurons and microglia derived from C57BL/6 mice to miR-100-5p, miR-298-5p (Fig. 5a-c), but also to miR-672-5p and let-7 g-5p for comparison (Additional file 8a, b)*.* In parallel, co-cultures were incubated with the mutated control oligonucleotide described above. It is well established that LPS and loxoribine induce neuronal injury through TLR4 and TLR7, respectively, expressed in microglia [6, 21, 22]. Therefore, these specific TLR agonists were included as positive controls in our approach. Immunocytochemical analysis revealed that exposure to miR-100-5p, miR-298-5p, and let-7g-5p led to a similar reduction in neuronal viability and concomitantly to an increase in the number of apoptotic cells (Fig. 5a-c, Additional file 8a, b). While exposure to miR-672-5p did not result in a significant loss of neurons, it induced an increase in apoptotic cell numbers (Additional file 8b). Exposure of co-cultures to LPS and loxoribine led to neuronal cell death, as expected (Fig. 5a-c, Additional file 8a, b).

Though TLRs are predominantly expressed in immune cells, neurons also express a few TLRs, including TLR7/8 [6, 8]. To test whether extracellular miR-298-5p and miR-100-5p enter neurons and localize to the endosomal compartment and/or TLR7, we incubated cortical neurons with the fluorescence-tagged oligoribonucleotides and conducted co-localization studies using confocal microscopy. Both miRNAs entered neurons within 4 h of miRNA exposure and co-localized to both endosomes and TLR7, as assessed by pHrodo Red Dextran (Additional file 9a) and TLR7 antibody (Additional file 9b) labeling, respectively. Based on these findings, we hypothesized that miR-298-5p and miR-100-5p trigger neurotoxicity in a cell-autonomous fashion. To test this, enriched cortical neurons were exposed to the respective miRNA or control mutant oligonucleotide, and neuronal viability was subsequently determined, as described above. LPS served as a negative control in this experimental set-up, as its cognate receptor TLR4, in contrast to microglia, is not expressed in neurons [21]. Thus, in the absence of relevant numbers of microglia, provided that neurons were sufficiently enriched, LPS does not induce neurotoxicity. miR-100-5p and miR-298-5p treatment of neurons resulted in reduced relative neuronal viability, and these neurotoxic effects were dose-dependent (Fig. 5d-f). miR-100-5p- and miR-298-5p-induced neurotoxicity required TLR7 signaling, as *Tlr7*^*−/−*^ neurons were protected from damage and cell loss (Fig. 5e). In comparison, let-7g-5p, but not miR-672-5p induced a reduction in relative neuronal viability dependent on TLR7 (Additional file 8c). Overall, the extent of miRNA-induced neuronal injury and loss observed in neuronal cell cultures with and without microglia did not significantly differ (see Fig. 5b, e, f and Additional file 8a, c). Thus, microglia were not required for neurotoxic effects in response to the tested miRNAs.

In conclusion, miR-100-5p, miR-298-5p, let-7g-5p, and to a lesser extent miR-672-5p, induced neuronal injury in vitro. This miRNA-induced neurotoxicity operated cell-autonomously through TLR7.

### miR-298-5p and miR-100-5p in cerebrospinal fluid trigger neurodegeneration in the cerebral cortex through TLR7

**Fig. 6:**
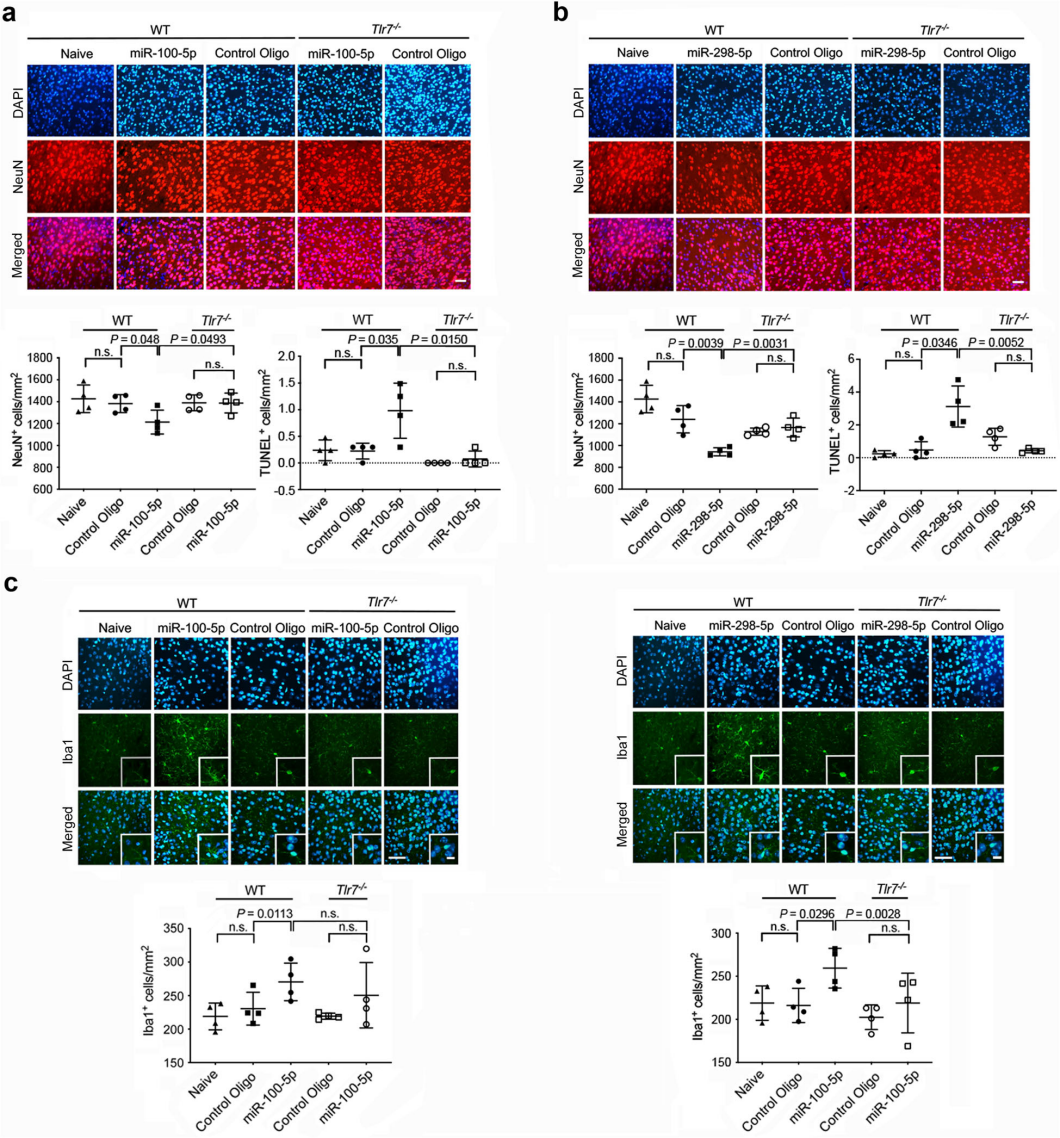
Intrathecal miR-298-5p and miR-100-5p induce neurodegeneration and microglial accumulation in a TLR7-dependent fashion. C57BL/6 (wild-type, WT) and *Tlr7*^*−/−*^ mice (*n* = 4 each group) received intrathecal injection of either 10 μg miR-100-5p (**a**, **c**), miR-298-5p (**b**, **d**), or 10 μg control mutant oligonucleotide (**a**-**d**). After 3 d, brain sections were immunolabeled with NeuN or Iba1 antibody, with DAPI, and with TUNEL assay. For comparison, brain sections of naïve WT mice were included in this experimental set-up. Representative images of brain sections labeled with NeuN antibody (upper panel), Iba1 antibody (lower panel), and DAPI are shown. Scale bar, 50 μm; inserts, scale bar, 10 μm. NeuN^+^ (upper panel, left graph), TUNEL^+^ (upper panel, right graph), and Iba1^+^ (lower panel, bottom graph) cells in the cerebral cortex were quantified. Data are shown as mean ± SD. *P* values for relevant groups as determined using the Student’s *t*-test are shown. n.s., not significant

To investigate the effects of miR-100-5p and miR-298-5p serving as direct TLR7 activators in the brain in vivo, we injected both wild-type (WT, C57BL/6) and *Tlr7*^*−/−*^ mice intrathecally with miR-100-5p, miR-298-5p, or mutant control oligonucleotide. Three days after injection, mice were sacrificed, and brain sections were analyzed by immunohistochemistry and TUNEL assay. Both miR-100-5p (Fig. 6a) and miR-298-5p (Fig. 6b) induced a reduction in neuronal density of the WT cerebral cortex. Consistent with these findings, analysis by TUNEL assay revealed an increase in apoptotic cell numbers in WT cortices of mice injected with miR-100-5p (Fig. 6a) and miR-298-5p (Fig. 6b) compared to control condition. Injection of mutant control oligonucleotide into WT mice did not affect neuronal density or rate of apoptosis in the cerebral cortex compared to naive animals, confirming sequence-specificity of the miR-100-5p- and miR-298-5p-mediated neurotoxic effects. Furthermore, *Tlr7*^*−/−*^ mice were protected against neurodegenerative effects induced by miR-100-5p and miR-298-5p (Fig. 6a, b). Microglia in the neocortex of miR-100-5p- and miR-298-5p-injected mice acquired an activated phenotype, while such a morphological change was not observed in control-injected WT or any of the injected *Tlr7*^*−/−*^ mice (Fig. 6c, d). Quantification of Iba1^+^ cells in the cerebral cortex revealed a 19.9% and 17.4% increase in microglial cell numbers of miR-298-5p- and miR-100-5p-injected WT mice compared to control, respectively. In contrast, brains of *Tlr7*^*−/−*^ mice did not display such effects (Fig. 6c, d).

Having observed neurodegenerative effects induced by synthetic miRNA, we sought to validate the potential of naturally occurring, brain-derived small RNA to induce neuronal damage in vivo. To this end, we performed intrathecal injections of small RNA isolated from mouse brain into mice. In this experimental set-up, we focused on miR-298-5p, which was identified as a potent TLR7 activator and inducer of neuroinflammatory and neurodegenerative effects in the experiments described above. In detail, small RNA (< 200 nt) was enriched from total RNA isolated from C57BL/6 cerebral cortices. C57BL/6 mice were intrathecally injected with a locked-nucleic acid (LNA)-based miR-298-5p inhibitor, a negative control inhibitor, or solvent. After 16 h, mice were injected for a second time with brain-derived small RNA. Sham-operated mice received 2 consequent injections with solvent. After a further 3 days, brains were analyzed by immunohistochemistry using a NeuN antibody (Additional file 10). Quantification of NeuN^+^ cells in the cerebral cortex of mice injected with solvent and small RNA revealed a 39.3% decrease in neuronal numbers in the cerebral cortex compared to sham-operated mice (*p* = 0.0003, Student’s *t* test). Consistently, neuronal nuclei in small RNA-treated mice appeared of irregular shape, shrunk, and fragmented, indicating neuronal cell death (Additional file 10). The reduction in neuronal numbers and changes in nucleic morphology were ameliorated in mice injected with small RNA and pre-treated with the miR-298-5p inhibitor (27.25% reduction in NeuN^+^ cell numbers compared to sham, *p* = 0.0001; solvent + small RNA vs. miR-298-5p inhibitor + small RNA, *p* = 0.0476, Student’s *t* test). Intrathecal injection of brain-derived small RNA following application of the negative control inhibitor did not result in such ameliorating effects, but resulted in similar neurodegenerative effects as observed in mice injected with small RNA without inhibitor treatment (solvent + small RNA vs. neg. control inhibitor + small RNA, *p* = 0.8376, Student’s *t* test; Additional file 10). Collectively, these data validate the potential of brain-derived small RNA, including miRNA miR-298-5p, to induce neurodegeneration in vivo.

To assess the impact of a neuroinflammatory environment on miRNA-induced injurious effects in vivo, we combined intrathecal miRNA injection into mice with LPS treatment. To this end, WT mice were intrathecally injected with LPS. After 16 h mice were injected a second time with miR-298-5p, miR-100-5p, or control mutant oligoribonucleotide (Additional file 11). Immunohistochemical analysis of the cerebral cortex after a further 3 d revealed that pre-treatment with LPS enhanced neurodegenerative effects of intrathecal miR-298-5p and miR-100-5p compared to the effects induced by the injection of the respective miRNA alone. In detail, intrathecal LPS plus miR-298-5p induced significant loss of neurons in the cerebral cortex, and this effect was enhanced by 12.7% compared to sole miR-298-5p injection (Additional file 11a, b). LPS plus miR-100-5p injection also resulted, although not reaching statistical significance, in enhanced neurodegenerative effects compared to sole miR-100-5p injection (12.6%; Additional file 11a, b). As expected, intrathecal LPS alone did not induce neuronal loss (Additional file 11a, b [53]). Cortical microglia in mice intrathecally injected with LPS only exhibited an activated phenotype and accumulated, as expected (Additional file 11c, d [53]). To analyze whether intrathecal miRNAs in combination with LPS treatment alter microglial numbers compared to sole miRNA injection, we quantified Iba1^+^ cells in the cerebral cortex. Injection of LPS plus miR-298-5p or miR-100-5p resulted in a 37% and 15.5% increase in microglial numbers, respectively, compared to mice that received the respective miRNA only (Additional file 11c, d). Injection of the control oligoribonucleotide into mice pre-treated with LPS resulted in an activated phenotype of microglia and microglial accumulation compared to the naïve condition, as expected (Additional file 11c, d).

In summary, extracellularly delivered miR-100-5p and miR-298-5p induced neurodegeneration and microglial accumulation in the cerebral cortex through TLR7 signaling in vivo. These effects were enhanced in an LPS-triggered neuroinflammatory state. Notably, while intrathecal LPS alone did not induce neurotoxicity, the combination with miR-100-5p or miR-298-5p resulted in distinct neurodegenerative effects in vivo. Our findings correlate with the results on miRNA-mediated toxicity on cortical neurons observed in vitro.

## Discussion

Using a systematic screening approach, we identified miRNAs that in extracellular form can serve as TLR ligands, and elaborated the consequences of such an interaction in CNS injury. To model the release of miRNAs from degenerating CNS neurons, we induced apoptosis in primary cortical neurons. Performing a miRNA GeneChip array we identified 39 miRNAs released upon apoptosis induction. We selected 12 out of these 39 miRNAs for further testing and found that six out of these 12 miRNAs activated human TLR7 and/or TLR8. The relevance of these findings for immune cell function was confirmed analyzing human-derived macrophages and murine microglia, which released cytokines and exhibited an increased phagocytic activity in response to the miRNAs. Remarkably, the miRNA species detected extracellularly overlap only to a small extent with those previously determined by small RNA sequencing [24]. In fact, only let-7d-5p and let-7e-5p were found being present in the extracellular space, conducting both approaches. Taking into account that different miRNA detection methods have their specific technical advantages and limitations, this discrepancy may highlight the need for the usage of complementary approaches to identify extracellular miRNAs, which in all probability are present in rather small amounts in a given tissue. While only a defined selection of the miRNAs released from apoptotic neurons was studied as potential TLR7/8 ligands in our study, our microarray screening approach provides 27 further miRNAs awaiting investigation with respect to their role as activators of TLRs or other receptor classes in future studies. Among the miRNAs detected in the supernatant of injured neurons and validated as TLR activators, were oligoribonuclotides such as miR-501-3p, exclusively activating human TLR7, whose role as signaling molecules in the brain was not further investigated in our study. In line with the assumption that a miRNA serving as a receptor ligand has to be present extracellularly in sufficient quantities, miR-501-3p is upregulated in brains of AD patients and is currently discussed as a potential biomarker for AD disease progression [54]. The data obtained from our miRNA microarray may be useful not only with respect to the miRNAs’ functional relevance in neurodegeneration, but may also serve as a valuable resource of candidate miRNAs potentially acting as receptor ligands in different pathologies. Our results suggest a regulated, rather than a non-specific release of miRNAs from injured CNS neurons and are in line with previous reports suggesting that extracellular miRNAs do not necessarily match the cellular miRNA origin [55]. However, our miRNA profiling of E17.5 cortical neurons provides information about the miRNA presence/expression at a specific stage of brain development and may be different at other stages. Also, apart from apoptotic neurons further cell populations in the CNS, e.g. microglia, may release miRNAs acting as endogenous TLR ligands, thereby potentially enabling intercellular communication.

To reveal key features of a particular miRNA being crucial for its function as a TLR7/8 ligand, we analyzed whether the secondary structure, potentially formed within a mature miRNA [44], has an impact on TLR activation. The minimal energy values calculated for select miRNAs identified by our miRNA microarray imply that the miRNA’s ability to activate TLR7/8 does not correlate with the probability of a mature miRNA to form stable secondary structure. Still, we cannot exclude that under certain conditions structural miRNA features providing molecular stability or enabling receptor binding might be required for an efficient interaction with TLRs.

Although individual miRNAs have been reported to activate TLRs in different cell types [6, 14, 15], the cellular and molecular mechanisms of their physical interaction with a given cell was largely unexplored. Using confocal microscopy we show that extracellular let-7b-5p, which was identified as direct TLR7 activator in our previous work [6], as well as the novel TLR7/8 ligands miR-298-5p and miR-100-5p, indeed enter both microglia and neurons, and localize to their endosomal compartment, as well as TLR7. Subsequent studies may clarify the detailed kinetics of the miRNAs’ entry and the molecular mechanisms of their trafficking to the endosomal compartment not only in microglia and neurons, but also in peripheral immune cells. In addition, further studies may decipher specific sequence motifs that allow the miRNA’s cellular uptake and trafficking. Also, whether miRNAs leak into the extracellular space in their native state, particularly as they are stable outside the cell [56], are engulfed within membranes, or/and form complexes with proteins [57], remains elusive at this stage. miRNAs linked to exosomes are released from lung cancer cells, thereby activating TLR signaling in immune cells [14], and it is conceivable that similar mechanisms exist in the CNS.

In order to be effective, miRNAs capable to directly activate receptors must engage their cognate receptor with sufficient affinity. Previous co-immunoprecipitation studies indicated that oligoribonucleotides released within exosomes bind to human TLR8 [14]. In sensory neurons, *let-7b* was shown to co-localize with TLR7 by immunolabeling [58]. However, it remained unclear whether the interaction between oligoribonucleotide and receptor was of direct or indirect nature. Here, we made use of microscale thermophoresis (MST) to determine whether miRNAs directly bind to human TLR8 protein, containing leucine-rich repeats (LRRs). The latter harbor the nucleic acid-sensing binding domains that are essential for TLR activation [59]. The dissociation constants obtained by MST indicate that miR-298-5p, miR-100-5p, let-7g-5p, and let-7b-5p indeed directly bind to human TLR8. Remarkably, although miR-100-5p activated both human TLR7 and TLR8, while miR-298-5p exclusively activated human TLR7, all of the tested miRNAs bound to human TLR8. This can be explained by previous studies focusing on the 3D structure of TLR7/8. Both TLRs possess two different ligand recognition sites within the LRRs. Co-activation of these sites by different RNA bases may act synergistically with respect to receptor activation [59–62]. In TLR7, the first binding site is preferentially occupied by guanosine (G), while the second site requires a base trimer with one uridine (U) [60]. However, while UU(C/U) provides full binding affinity to the second TLR7 binding site, UU(G/A) only moderately binds to the same site [63]. For TLR8 activation, the first binding site requires binding of U, while the second binding site needs binding of UG [60]. Since the sequence of miR-298-5p contains several G and U moieties, it may dock to specific binding sites present in both human TLR7 and TLR8. Unique activation of TLR7 by miR-298-5p is most likely achieved by the prominent presence of G and the additional UUCUUC motif in the mature sequence, whereas the presence of only one UG (see Table 1) may not be sufficient to activate human TLR8. We found that a control oligoribonucleotide with a mutated sequence exhibited much lower binding affinity to TLR8 compared to the other tested miRNAs and, consequently, failed to trigger a cellular response. Thus, the effects of the miRNAs identified as TLR ligands were sequence-specific, and the respective miRNA sequence alone may provide sufficient regulation of receptor activation/inhibition.

In human postmortem brain, RNA is present extracellularly and associated with senile plaques [64]. Expression of miR-100-5p, which we here present as a novel human TLR7/8 ligand, was previously reported to be upregulated in frontal gyrus and cerebellum of AD patients [46]. Likewise, expression of miR-100-5p, but also let-7b-5p, is upregulated in the cerebral cortex of APP/PS1 mice, a model for AD [50, 65]. Consistent with this, miR-100-5p and *let-7b* copies in AD cerebrospinal fluid (CSF) are specifically increased, leading to the discussion whether these miRNAs can serve as biomarkers for AD [17, 47]. miR-298-5p, also identified as novel TLR7 activator that contributes to neuronal damage in our study, has also been previously linked to AD [48, 49]. Especially, miR-298-5p transcript levels in temporal lobes of AD patients were increased [49]. Altogether, these data support our data derived from injected mice that extracellular miRNAs such as miR-100-5p and miR-298-5p can contribute to neurodegeneration through direct receptor action. Moreover, to the best of our knowledge our studies show for the first time that intrathecal brain-derived small RNA, including miRNA miR-298-5p, is capable of triggering neurodegenerative effects in vivo. In addition, miRNA-induced neuronal injury and microglial accumulation were enhanced in an LPS-triggered neuroinflammatory setting. While intrathecal LPS alone did not induce neurotoxicity, the combination with miR-100-5p or miR-298-5p resulted in distinct neurodegeneration. These findings point to a complex interplay between extracellular miRNAs, further molecule classes modulating the inflammatory state in the brain, and different TLRs, such as TLR7 and TLR4. Specific CNS disease mouse models, such as AD mice, should be used to investigate the role of miRNAs acting as signaling molecules in the pathogenesis and progress of neurodegenerative and neuroinflammatory diseases in future studies. Moreover, the potential value of these miRNAs in diagnosis and treatment of neurodegenerative diseases such as AD may be evaluated herein. CNS disease mouse models may also be suitable to visualize the uptake of extracellular miRNAs by different brain cell types and to study its underlying molecular mechanisms in vivo. In addition, to gain deeper insight into the complex role of microglia and the neuroinflammatory response in miRNA-triggered neurodegeneration, further in vivo studies under microglia-depleted conditions are required.

Apart from TLR7 and TLR8 [6, 8], several other TLRs are expressed in neurons under certain conditions [66–68]. These TLRs, among other receptor classes, may also bind to miRNAs and serve as death receptors. Also, as several miRNAs acting as TLR ligands induced neurotoxicity, but did not affect the viability of microglia or macrophages in this study (data not shown), the responding cell type may determine the specific outcome of the miRNA-TLR7/8 interaction.

At this stage the amount of miRNA species effectively released in brain hemostasis or at the site of brain injury in a neurodegenerative context remains unclear, as does the local miRNA concentration. The lowest concentration that induced hTLR8 activation was 0.1 μg/ml of miR-100-5p (13.48 nM). In HEK hTLR7 reporter cells, 1 μg/ml of miR-298-5p (127.75 nM) led to significant activation. In mouse microglia, doses of miR-100-5p and miR-298-5p as low as 0.1 μg/ml (13.48 nM and 12.77 nM, respectively) induced cytokine release within the tested time period. Based on i) the total yield of about 160 ng (22.89 nM) of miRNAs released from 2 million neurons into neuronal medium (see Fig. 1), and ii) assuming an average molecular weight of 7000 g/mol for a miRNA copy according to the Avogadro constant [69], we calculated that each cortical neuron can release about 6.9 millions of miRNA copies. Given that TLR7/8 and microglia in principle can be activated by miRNA concentrations of about 12–14 nM (see above) and considering an extracellular accumulation of (specific) miRNAs during a chronic disease progress over an extended time period [17, 70], miRNA concentrations may well reach levels allowing receptor activation under pathophysiological conditions. However, it remains unresolved whether miRNA present in CSF can reach physiological concentrations to activate membrane receptors, such as TLR7/8. Also, whether such miRNA concentrations in CSF correlate with the local concentrations within the brain parenchyma, especially in the setting of neurodegeneration, is unclear. Thus, we consider our experiments on neuronal injury and microglial activation induced by the novel TLR7/8 miRNA ligands released from apoptotic CNS neurons as a proof-of-principle study, and we cannot rule out that supraphysiological miRNA doses were used. Future work will be required to determine the pathophysiological concentrations of extracellular miRNAs and their functional relevance in brain health and disease.

## Conclusions

We demonstrate here that apoptotic cortical neurons release miRNAs that act as endogenous TLR7 and/or TLR8 ligands. As a consequence, these miRNAs contribute to CNS inflammation and neurodegeneration. Our data further extend the role of miRNAs beyond gene expression regulation to ligand-mediated receptor activation. In this manner, miRNAs may modulate not only immune responses and neurodegenerative processes in the brain, but also affect other organ systems. Thus, our study provides a basis for future investigations of molecular and mechanistic aspects of the interaction between miRNAs and TLRs in various human disorders.

## Supplementary Information

**Additional file 1.** Quality control parameter after normalization for mouse probes of the GeneChip miRNA array. Density histograms of probe intensities (a) and principal components analysis (b) for apoptotic cortical neurons and corresponding neuronal supernatant (S/N), as indicated, are shown.

**Additional file 2. **Amounts of miRNAs released from apoptotic cortical neurons do not correlate with the abundance of miRNAs expressed in control neurons. (a) Scatter plot of Log2 Fold Change of miRNAs being enriched in supernatant (S/N) derived from staurosporine-treated neurons (*P* < 0.01, Log2 Fold Change > 1: 39 miRNAs) plotted against their intracellular concentration in control neurons treated with 0.1% DMSO as solvent. (b) Scatter plot of Log2 Fold Change miRNAs being enriched in S/N of staurosporine-treated neurons (*P* < 0.05; 88 miRNAs) plotted against their intracellular concentration in control neurons treated with 0.1% DMSO as solvent. In (a) and (b), the respective Pearson correlation coefficient (r) is depicted.

**Additional file 3.** Gene Ontology (GO) enrichment analysis of biological processes for miRNAs that are enriched in supernatant of apoptotic cortical neurons. Color refers to significance (FDR, false discovery rate), while size indicates the corresponding number of associated genes. S/N, supernatant.

**Additional file 4. **miR-672-5p, *let-7g*-5p, miR-100-5p, and miR-298-5p induce TNF-α release from microglia in a time- and dose-dependent fashion. (a) C57BL/6 (wild-type, WT) microglia were incubated with 5 μg/ml of miRNA, as indicated, for indicated durations. (b) WT microglia were incubated with various doses of indicated miRNAs for 24 h followed by TNF-α ELISA. In all experiments (a, b), the TLR7 agonist loxoribine (1 mM) and the TLR4 agonist LPS (100 ng/ml) served as positive control. Unstimulated condition served as negative control. Data are represented as mean ± SD, *n* = 3.

**Additional file 5. **Low dose response of miR-100-5p- and miR-298-5p-treated microglia and HEK293 hTLR7/8 reporter cells. (a) Images of C57BL/6 microglia incubated with 5 μg/ml of miR-100-5p or miR-298-5p, or PBS (control) for 4 h. Subsequently, cells were fixed and immunolabeled with Iba1 antibody, while nuclei were visualized with DAPI. Compared to control, miRNA-treated microglia displayed an amoeboid morphology, indicating an activated state. Scale bar, 30 μm. (b) Microglia were incubated with indicated doses of miR-100-5p or miR-298-5p for 24 h. Loxoribine (1 mM) and LPS (100 ng/ml) served as positive control. Unstimulated condition served as negative control. Subsequently, supernatants were analyzed by TNF-α ELISA. Data are represented as mean ± SD, *n* = 3. (**c**) HEK-Blue cells co-expressing human TLR7 (left, center) or human TLR8 (right), and an NF-κB/AP1-inducible secreted embryonic alkaline phosphatase (SEAP) reporter gene were incubated with various doses of miR-100-5p or miR-298-5p, as indicated, for 24 h. Loxoribine (1 mM), R848 (100 ng/ml), or TNF-α (100 ng/ml) served as positive control. Unstimulated HEK-Blue TLR-expressing cells and HEK-Blue Null1 or Null1-k cells served as negative control. Data are expressed as fold change of optical density of the SEAP protein normalized to unstimulated control. Data are represented as mean ± SD, *n* = 3. *P < 0.05 compared to the unstimulated condition, Student’s *t*-test.

**Additional file 6. **Extracellular miR-100-5p and miR-298-5p increase microglial phagocytic activity. Microglia from C57BL/6 mice were exposed to 5 μg/ml of the indicated miRNAs, LPS (100 ng/ml), or solvent control (PBS). After 2 h, microglia were incubated with red fluorescent beads of 1 μm size for 1 h. Subsequently, cells were labeled with Iba1 antibody, and nuclei were visualized with DAPI (Scale bar, 30 μm, left). Bead-linked red fluorescence within Iba1-positive image areas was quantified using FiJi software. Fluorescence intensity (FI) was expressed in arbitrary units (a.u., right). Data are represented as mean ± SD. *n* = 3. *P < 0.05; **P < 0.01 compared to control, Student’s *t*-test.

**Additional file 7. **Microscale thermophoresis measurement of let-7g-5p and specificity control. Binding affinity measurements of the purified polyhistidine-tagged human TLR8 protein and (a) let-7g-5p and (b) miR-298-5p using microscale thermophoresis (MST). (b) The hTLR8 protein fragment was incubated with the control peptide before start of the measurement. (a, b) TLR8-let-7g-5p and TLR8-miR-298-5p interaction was monitored by titrating oligonucleotides from 500 μM to 30 nM against 50 nM RED-tris-NTA-labeled hTLR8-protein measured with the NanoTemper Monolith NT.115 MST device. *K*_*d*_ values were calculated from dose response curves, which were obtained from titration experiments (*n* = 4). Data are expressed as mean ± SD.

**Additional file 8. **let-7g-5p and miR-672-5p induce neuronal injury in vitro. (a, b) Co-cultures of C57BL/6 (wild-type, WT) microglia and neurons were incubated with 5 μg/ml of indicated miRNAs for 5 d. Mutant control oligonucleotide and unstimulated cells were used as negative control. Cells were subsequently immunostained with NeuN antibody and stained with TUNEL assay and DAPI. Quantification of NeuN (depicted as relative neuronal viability, left)- and TUNEL (right)-positive cells in co-cultures. Data are expressed as mean ± SD, *n* = 4. *P < 0.05; **P < 0.01; ***P < 0.001 compared to unstimulated condition, Student’s *t*-test. (c) Enriched WT and *Tlr7*^*−/−*^ cortical neurons were incubated with 5 μg/ml of indicated miRNAs for 5 d. Cell cultures were subsequently immunostained with NeuN antibody and with DAPI. LPS (100 ng/ml) was used to test for potential relevant contamination of enriched neuronal cell cultures with microglia. Mutant control oligonucleotide and unstimulated cells were used as negative control. NeuN-positive neurons were quantified, and data are depicted as relative neuronal viability of cells treated with the indicated miRNA compared to control. Results are shown as mean ± SD (*n* = 4 for WT, *n* = 3 for *Tlr7*^*−/−*^ neurons). *P* value as indicated compared to unstimulated condition, Student’s *t*-test.

**Additional file 9.** miR-298-5p and miR-100-5p enter neurons and co-localize to their endosomal compartment and TLR7. (a) Enriched C57BL/6 cortical neurons were incubated with 40 μg/ml pHrodo Red Dextran serving as endosomal marker for 20 min. Subsequently, neurons were exposed to 5 μg/ml of Alexa488-labeled miR-298-5p or Alexa488-labeled miR-100-5p, and fixed after 4 h. Scale bar, 10 μm. (b) Neurons exposed to the fluorescence-tagged miRNAs, as described above, were fixed and immunolabeled with TLR7 antibody. Scale bar, 20 μm. (a, b) Cells were analyzed by confocal microscopy with sequential analysis. Representative images of neurons incubated with the indicated fluorescent miRNAs (488 nm, green) and pHrodo Red Dextran or TLR7 (552 nm, red) are shown (left panel). Diagrams depict fluorescence intensities of the marked ROI in neurons for the sequential analysis used (pHrodo Red Dextran/TLR7: red line; fluorescent miRNA: green line, right panel).

**Additional file 10. **Intrathecal brain-derived, small RNA triggers neurodegeneration. 125 pmol of an LNA inhibitor specific for mmu-miR-298-5p (miR-298-I), an LNA negative control inhibitor (neg. co.-I), or solvent (H_2_O) were injected intrathecally into C57BL/6 mice. After 16 h, mice were injected intrathecally for a second time with 10 μg of small RNA enriched from C57BL/6 mouse brain RNA, or solvent (solvent + solvent (sham), *n* = 4; solvent + small RNA (smRNA), *n* = 4; miR-298-5p inhibitor + small RNA (miR-298-I + smRNA), *n* = 4; neg. control inhibitor + small RNA (neg. co.-I + smRNA), *n* = 4). After a further 3 d, brain sections were immunolabeled with a NeuN antibody, and with DAPI. (a) Representative images of brain sections labeled with NeuN antibody and DAPI are shown. Scale bar, 50 μm; insets, scale bar, 10 μm. (b) NeuN^+^ cells in the cerebral cortex were quantified. Data are shown as mean ± SD. *P* values for relevant groups as determined using the Student’s *t*-test are shown. n.s., not significant.

**Additional file 11. **Intrathecal pre-treatment with LPS enhances miR-298-5p- and miR-100-5p-induced neurodegeneration and microglial accumulation. 1 μg LPS was injected intrathecally into C57BL/6 mice. After 16 h, mice were injected intrathecally with 10 μg of miR-298-5p, miR-100-5p, or control oligoribonucleotide (LPS alone, *n* = 4; LPS + control oligo, *n* = 4; LPS + miR-298-5p, *n* = 4; LPS + miR-100-5p, *n* = 4). Naive mice and mice solely injected with control oligoribonucleotide, miR-100-5p, or miR-298-5p were included in this experimental set-up (naive, *n* = 4; control oligo, *n* = 4; miR-298-5p, *n* = 4; miR-100-5p, *n* = 4). After 3 d, brain sections were immunostained with NeuN or Iba1 antibody, and with DAPI. Representative images of brain sections labeled with NeuN antibody (a), Iba1 antibody (c), and DAPI (a, c) are shown. Scale bar, 50 μm; inserts, scale bar, 10 μm. NeuN^+^ (b) and Iba1^+^ (d) cells in the cerebral cortex were quantified. Data are shown as mean ± SD. *P* values for relevant groups as determined using the Student’s *t*-test are shown in (b) and (d). n.s., not significant.

**Additional file 12: Table S1.** miRNA microarray information.

**Additional file 13: Table S2.** miRNA concentrations used in this study given in [nm].

## Data Availability

The datasets used and/or analyzed during the current study are available from the corresponding author on reasonable request.

## Abbreviations

AD: Alzheimer’s disease
CNS: Central nervous system
DAMP: Damage-associated molecular pattern
DMSO: Dimethyl sulfoxide
ELISA: Enzyme-linked immunosorbent assay
G: Guanosine
GO: Gene ontology
His-Tag: Polyhistidine-tagged
IFN: Interferon
IL: Interleukin
LPS: Lipopolysaccharide
MCP: Monocyte chemoattractant protein
MIP: Macrophage inflammatory protein
miRNA: microrna
MST: Microscale thermophoresis
NeuN: Neuronal nuclei
NF-κB: Nuclear factor ‘kappa-light-chain-enhancer’ of activated B-cells
RED-tris-NTA: RED-tris-nitrilotriacetic acid
SEAP: Secreted embryonic alkaline phosphatase
S/N: Supernatant
ssRNA: Single-stranded RNA
TLR: Toll-like receptor
TNF: Tumor necrosis factor
TUNEL: TdT-mediated dUTP-biotin nick end labeling
U: Uridine
