# Supplementary material for: MicroRNA-100-5p and microRNA-298-5p released from apoptotic cortical neurons are endogenous Toll-like receptor 7/8 ligands that contribute to neurodegeneration
Source: Mol Neurodegener. 2021 Nov 27;16:80. doi: 10.1186/s13024-021-00498-5 (PMC8626928; doi:10.1186/s13024-021-00498-5)
Supplement: Supplementary file 1 — Additional file 1. Quality control parameter after normalization for mouse probes of the GeneChip miRNA array. Density histograms of probe intensities (a) and principal components analysis (b) for apoptotic cortical neurons and corresponding neuronal supernatant (S/N), as indicated, are shown. [file 13024_2021_498_MOESM1_ESM.pdf]

Additional files

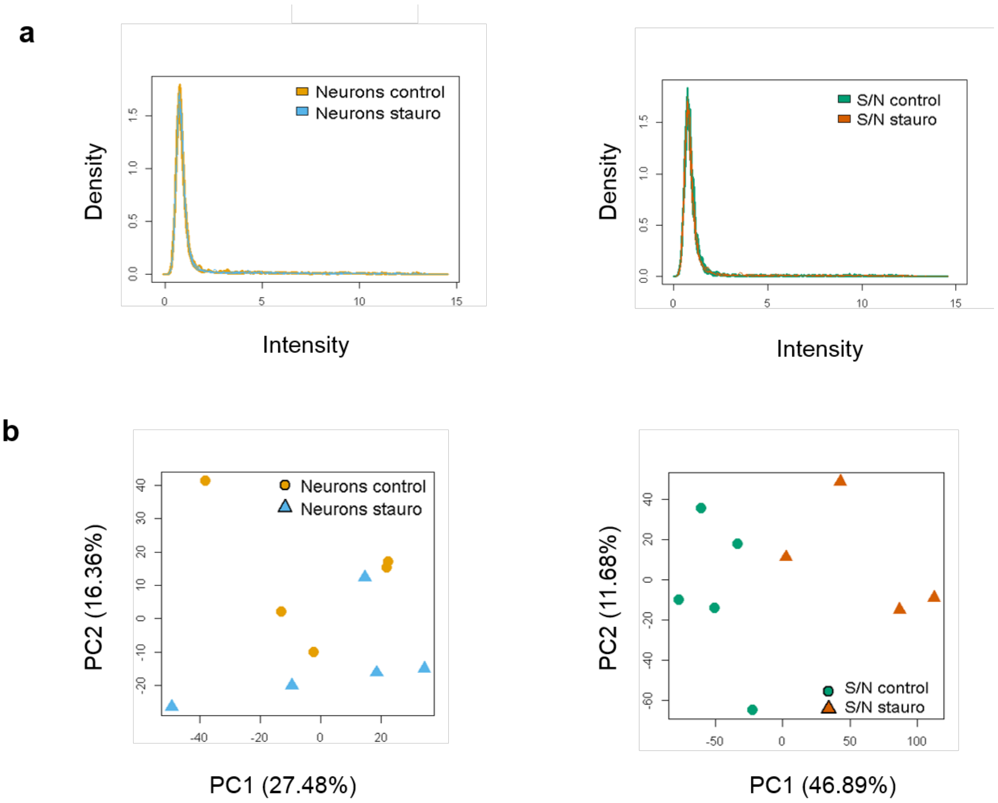

Additional file 1 Quality control parameter after normalization for mouse probes of the GeneChip miRNA array. Density histograms of probe intensities (**a**) and principal components analysis (**b**) for apoptotic cortical neurons and corresponding neuronal supernatant (S/N), as indicated, are shown.
