# Supplementary material for: MicroRNA-100-5p and microRNA-298-5p released from apoptotic cortical neurons are endogenous Toll-like receptor 7/8 ligands that contribute to neurodegeneration
Source: Mol Neurodegener. 2021 Nov 27;16:80. doi: 10.1186/s13024-021-00498-5 (PMC8626928; doi:10.1186/s13024-021-00498-5)
Supplement: Supplementary file 2 — Additional file 2. Amounts of miRNAs released from apoptotic cortical neurons do not correlate with the abundance of miRNAs expressed in control neurons. (a) Scatter plot of Log2 Fold Change of miRNAs being enriched in supernatant (S/N) derived from staurosporine-treated neurons (P < 0.01, Log2 Fold Change > 1: 39 miRNAs) plotted against their intracellular concentration in control neurons treated with 0.1% DMSO as solvent. (b) Scatter plot of Log2 Fold Change miRNAs being enriched in S/N of staurosporine-treated neurons (P < 0.05; 88 miRNAs) plotted against their intracellular concentration in control neurons treated with 0.1% DMSO as solvent. In (a) and (b), the respective Pearson correlation coefficient (r) is depicted. [file 13024_2021_498_MOESM2_ESM.pdf]

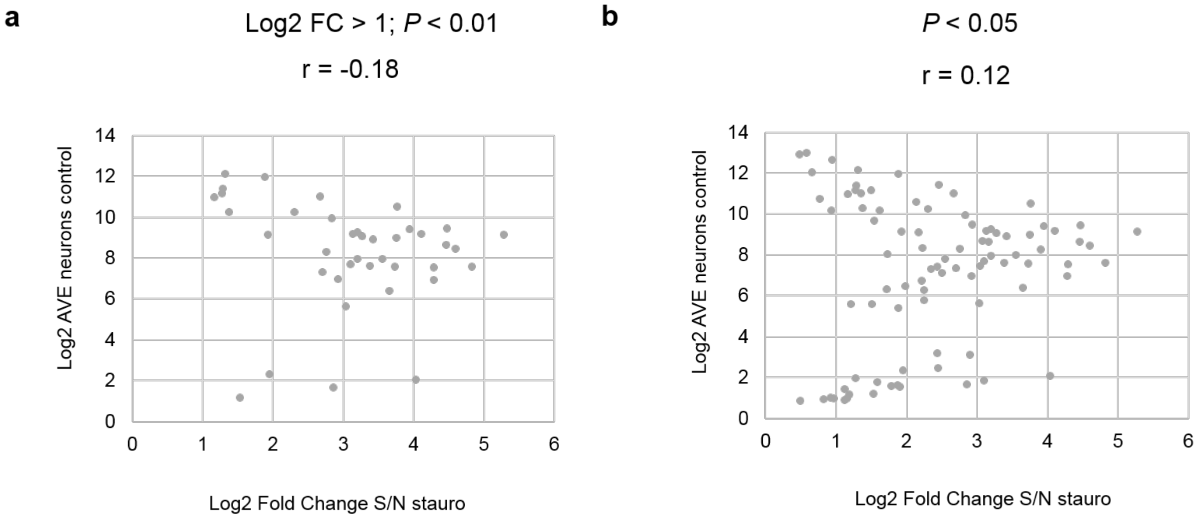

Additional file 2 Amounts of miRNAs released from apoptotic cortical neurons do not correlate with the abundance of miRNAs expressed in control neurons. (**a**) Scatter plot of Log2 Fold Change of miRNAs being enriched in supernatant (S/N) derived from staurosporine-treated neurons ($P < 0.01$, Log2 Fold Change > 1: 39 miRNAs) plotted against their intracellular concentration in control neurons treated with 0.1% DMSO as solvent. (**b**) Scatter plot of Log2 Fold Change miRNAs being enriched in S/N of staurosporine-treated neurons ($P < 0.05$; 88 miRNAs) plotted against their intracellular concentration in control neurons treated with 0.1% DMSO as solvent. In (**a**) and (**b**), the respective Pearson correlation coefficient (r) is depicted.
