# Supplementary material for: MicroRNA-100-5p and microRNA-298-5p released from apoptotic cortical neurons are endogenous Toll-like receptor 7/8 ligands that contribute to neurodegeneration
Source: Mol Neurodegener. 2021 Nov 27;16:80. doi: 10.1186/s13024-021-00498-5 (PMC8626928; doi:10.1186/s13024-021-00498-5)
Supplement: Supplementary file 3 — Additional file 3. Gene Ontology (GO) enrichment analysis of biological processes for miRNAs that are enriched in supernatant of apoptotic cortical neurons. Color refers to significance (FDR, false discovery rate), while size indicates the corresponding number of associated genes. S/N, supernatant. [file 13024_2021_498_MOESM3_ESM.pdf]

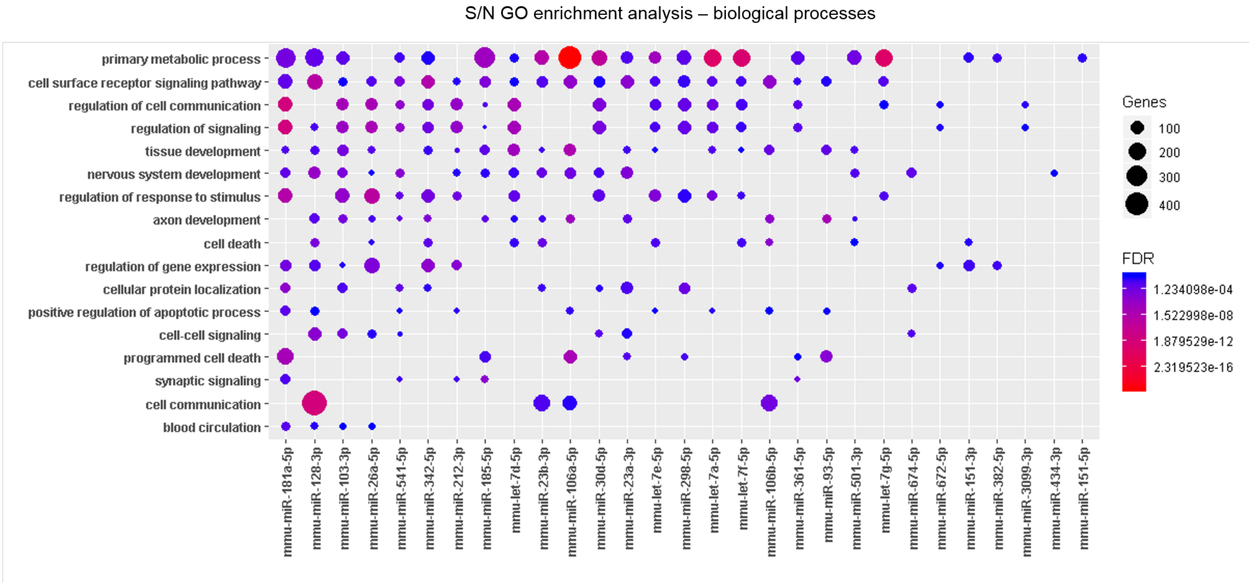

Additional file 3 Gene Ontology (GO) enrichment analysis of biological processes for miRNAs that are enriched in supernatant of apoptotic cortical neurons. Color refers to significance (FDR, false discovery rate), while size indicates the corresponding number of associated genes. S/N, supernatant.
