# Supplementary material for: MicroRNA-100-5p and microRNA-298-5p released from apoptotic cortical neurons are endogenous Toll-like receptor 7/8 ligands that contribute to neurodegeneration
Source: Mol Neurodegener. 2021 Nov 27;16:80. doi: 10.1186/s13024-021-00498-5 (PMC8626928; doi:10.1186/s13024-021-00498-5)
Supplement: Supplementary file 4 — Additional file 4. miR-672-5p, let-7g-5p, miR-100-5p, and miR-298-5p induce TNF-α release from microglia in a time- and dose-dependent fashion. (a) C57BL/6 (wild-type, WT) microglia were incubated with 5 μg/ml of miRNA, as indicated, for indicated durations. (b) WT microglia were incubated with various doses of indicated miRNAs for 24 h followed by TNF-α ELISA. In all experiments (a, b), the TLR7 agonist loxoribine (1 mM) and the TLR4 agonist LPS (100 ng/ml) served as positive control. Unstimulated condition served as negative control. Data are represented as mean ± SD, n = 3. [file 13024_2021_498_MOESM4_ESM.pdf]

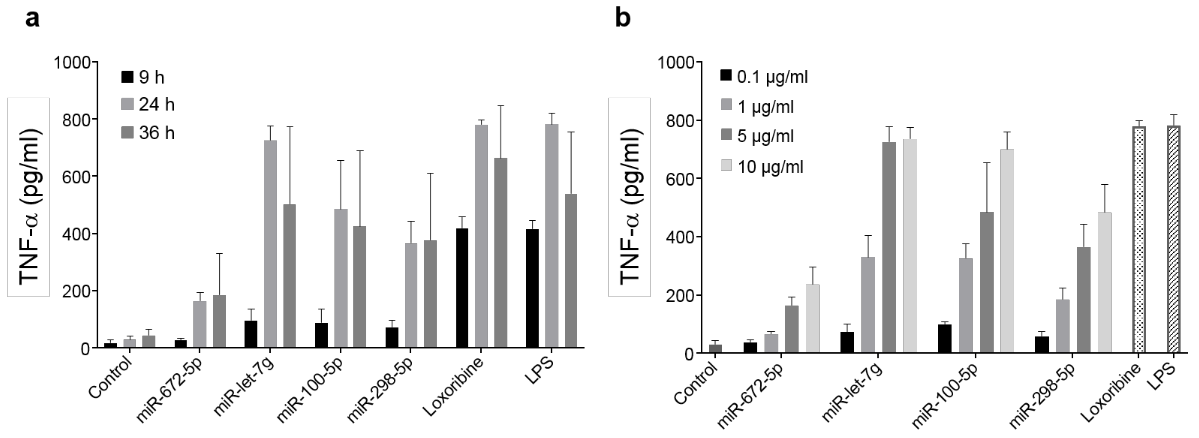

Additional file 4 miR-672-5p, *let-7g*-5p, miR-100-5p, and miR-298-5p induce TNF-α release from microglia in a time- and dose-dependent fashion. (**a**) C57BL/6 (wild-type, WT) microglia were incubated with 5 µg/ml of miRNA, as indicated, for indicated durations. (**b**) WT microglia were incubated with various doses of indicated miRNAs for 24 h followed by TNF-α ELISA. In all experiments (**a**, **b**), the TLR7 agonist loxoribine (1 mM) and the TLR4 agonist LPS (100 ng/ml) served as positive control. Unstimulated condition served as negative control. Data are represented as mean +/- SD, *n* = 3.
