# Supplementary material for: MicroRNA-100-5p and microRNA-298-5p released from apoptotic cortical neurons are endogenous Toll-like receptor 7/8 ligands that contribute to neurodegeneration
Source: Mol Neurodegener. 2021 Nov 27;16:80. doi: 10.1186/s13024-021-00498-5 (PMC8626928; doi:10.1186/s13024-021-00498-5)
Supplement: Supplementary file 5 — Additional file 5. Low dose response of miR-100-5p- and miR-298-5p-treated microglia and HEK293 hTLR7/8 reporter cells. (a) Images of C57BL/6 microglia incubated with 5 μg/ml of miR-100-5p or miR-298-5p, or PBS (control) for 4 h. Subsequently, cells were fixed and immunolabeled with Iba1 antibody, while nuclei were visualized with DAPI. Compared to control, miRNA-treated microglia displayed an amoeboid morphology, indicating an activated state. Scale bar, 30 μm. (b) Microglia were incubated with indicated doses of miR-100-5p or miR-298-5p for 24 h. Loxoribine (1 mM) and LPS (100 ng/ml) served as positive control. Unstimulated condition served as negative control. Subsequently, supernatants were analyzed by TNF-α ELISA. Data are represented as mean ± SD, n = 3. (c) HEK-Blue cells co-expressing human TLR7 (left, center) or human TLR8 (right), and an NF-κB/AP1-inducible secreted embryonic alkaline phosphatase (SEAP) reporter gene were incubated with various doses of miR-100-5p or miR-298-5p, as indicated, for 24 h. Loxoribine (1 mM), R848 (100 ng/ml), or TNF-α (100 ng/ml) served as positive control. Unstimulated HEK-Blue TLR-expressing cells and HEK-Blue Null1 or Null1-k cells served as negative control. Data are expressed as fold change of optical density of the SEAP protein normalized to unstimulated control. Data are represented as mean ± SD, n = 3. *P < 0.05 compared to the unstimulated condition, Student’s t-test. [file 13024_2021_498_MOESM5_ESM.pdf]

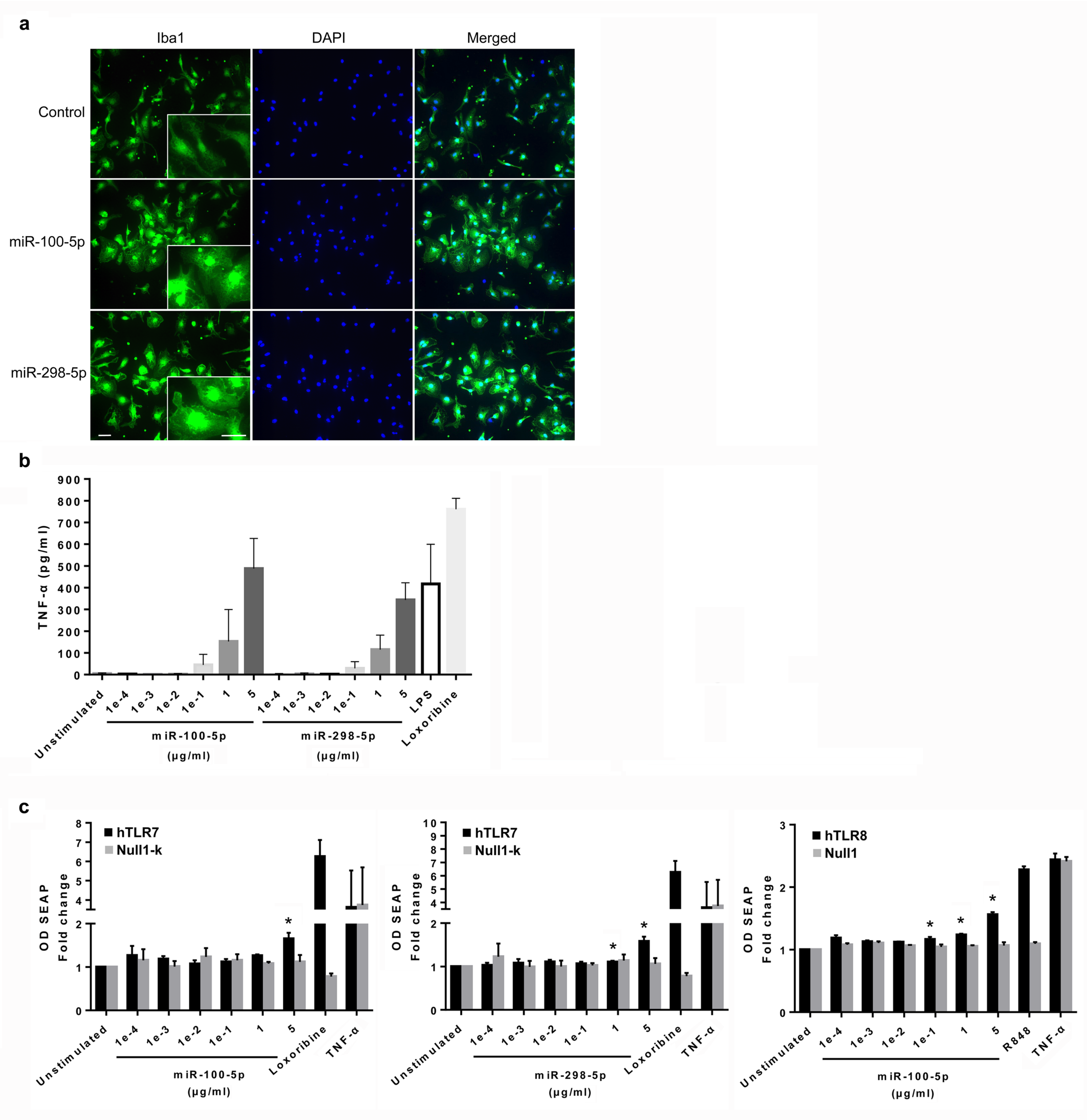

Additional file 5 Low dose response of miR-100-5p- and miR-298-5p-treated microglia and HEK293 hTLR7/8 reporter cells. (**a**) Images of C57BL/6 microglia incubated with 5 µg/ml of miR-100-5p or miR-298-5p, or PBS (control) for 4 h. Subsequently, cells were fixed and immunolabeled with Iba1 antibody, while nuclei were visualized with DAPI. Compared to control, miRNA-treated microglia displayed an amoeboid morphology, indicating an activated state. Scale bar, 30 µm. (**b**) Microglia were incubated with indicated doses of miR-100-5p or miR-298-5p for 24 h.

Loxoribine (1 mM) and LPS (100 ng/ml) served as positive control. Unstimulated condition served as negative control. Subsequently, supernatants were analyzed by TNF-α ELISA. Data are represented as mean±SD, *n* = 3. (**c**) HEK-Blue cells co-expressing human TLR7 (left, center) or human TLR8 (right), and an NF-κB/AP1-inducible secreted embryonic alkaline phosphatase (SEAP) reporter gene were incubated with various doses of miR-100-5p or miR-298-5p, as indicated, for 24 h. Loxoribine (1 mM), R848 (100 ng/ml), or TNF-α (100 ng/ml) served as positive control. Unstimulated HEK-Blue TLR-expressing cells and HEK-Blue Null1 or Null1-k cells served as negative control. Data are expressed as fold change of optical density of the SEAP protein normalized to unstimulated control. Data are represented as mean±SD, *n* = 3. **P* < 0.05 compared to the unstimulated condition, Student's *t*-test.
