# Supplementary material for: MicroRNA-100-5p and microRNA-298-5p released from apoptotic cortical neurons are endogenous Toll-like receptor 7/8 ligands that contribute to neurodegeneration
Source: Mol Neurodegener. 2021 Nov 27;16:80. doi: 10.1186/s13024-021-00498-5 (PMC8626928; doi:10.1186/s13024-021-00498-5)
Supplement: Supplementary file 6 — Additional file 6. Extracellular miR-100-5p and miR-298-5p increase microglial phagocytic activity. Microglia from C57BL/6 mice were exposed to 5 μg/ml of the indicated miRNAs, LPS (100 ng/ml), or solvent control (PBS). After 2 h, microglia were incubated with red fluorescent beads of 1 μm size for 1 h. Subsequently, cells were labeled with Iba1 antibody, and nuclei were visualized with DAPI (Scale bar, 30 μm, left). Bead-linked red fluorescence within Iba1-positive image areas was quantified using FiJi software. Fluorescence intensity (FI) was expressed in arbitrary units (a.u., right). Data are represented as mean ± SD. n = 3. *P < 0.05; **P < 0.01 compared to control, Student’s t-test. [file 13024_2021_498_MOESM6_ESM.pdf]

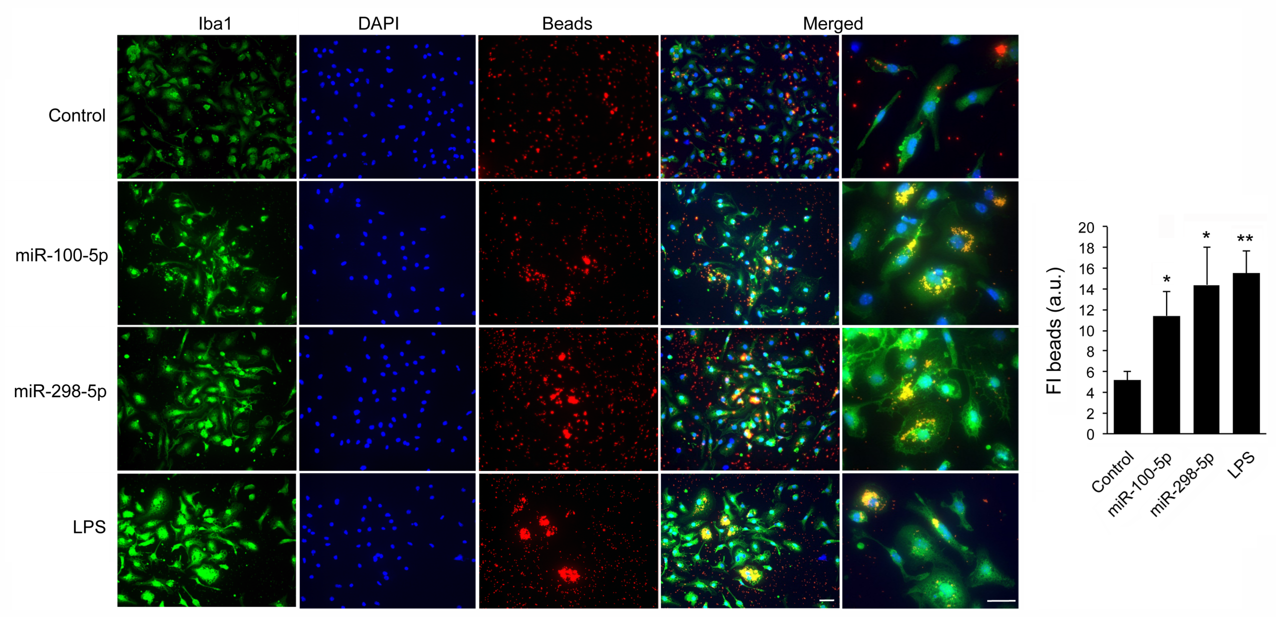

Additional file 6 Extracellular miR-100-5p and miR298-5p increase microglial phagocytic activity. Microglia from C57BL/6 mice were exposed to 5 µg/ml of the indicated miRNAs, LPS (100 ng/ml), or solvent control (PBS). After 2 h, microglia were incubated with red fluorescent beads of 1 µm size for 1 h. Subsequently, cells were labeled with Iba1 antibody, and nuclei were visualized with DAPI (Scale bar, 30 µm, left). Bead-linked red fluorescence within Iba1-positive image areas was quantified using FiJi software. Fluorescence intensity (FI) was expressed in arbitrary units (a.u., right). Data are represented as mean±SD. $n = 3$. *$P < 0.05$; **$P < 0.01$ compared to control, Student's *t*-test.
