# Supplementary material for: MicroRNA-100-5p and microRNA-298-5p released from apoptotic cortical neurons are endogenous Toll-like receptor 7/8 ligands that contribute to neurodegeneration
Source: Mol Neurodegener. 2021 Nov 27;16:80. doi: 10.1186/s13024-021-00498-5 (PMC8626928; doi:10.1186/s13024-021-00498-5)
Supplement: Supplementary file 7 — Additional file 7. Microscale thermophoresis measurement of let-7g-5p and specificity control. Binding affinity measurements of the purified polyhistidine-tagged human TLR8 protein and (a) let-7g-5p and (b) miR-298-5p using microscale thermophoresis (MST). (b) The hTLR8 protein fragment was incubated with the control peptide before start of the measurement. (a, b) TLR8-let-7g-5p and TLR8-miR-298-5p interaction was monitored by titrating oligonucleotides from 500 μM to 30 nM against 50 nM RED-tris-NTA-labeled hTLR8-protein measured with the NanoTemper Monolith NT.115 MST device. Kd values were calculated from dose response curves, which were obtained from titration experiments (n = 4). Data are expressed as mean ± SD. [file 13024_2021_498_MOESM7_ESM.pdf]

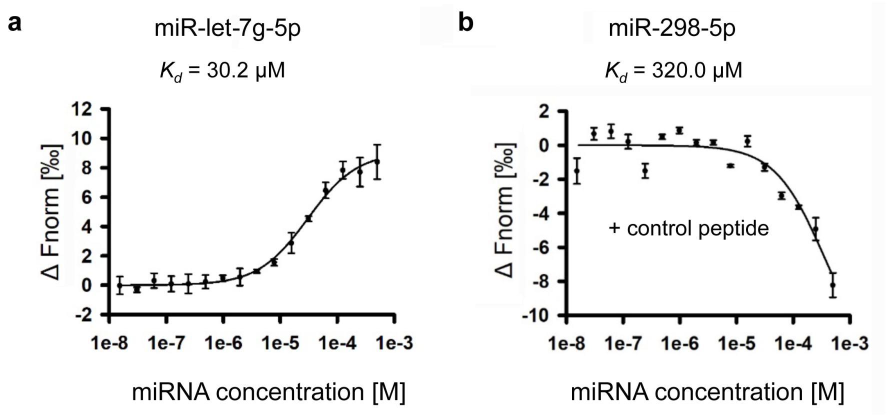

Additional file 7 Microscale thermophoresis measurement of let-7g-5p and specificity control. Binding affinity measurements of the purified polyhistidine-tagged human TLR8 protein and (**a**) let-7g-5p and (**b**) miR-298-5p using microscale thermophoresis (MST). (**b**) The hTLR8 protein fragment was incubated with the control peptide before start of the measurement. (**a**, **b**) TLR8-let-7g-5p and TLR8-miR-298-5p interaction was monitored by titrating oligonucleotides from 500 µM to 30 nM against 50 nM RED-tris-NTA-labeled hTLR8-protein measured with the NanoTemper Monolith NT.115 MST device. K_d values were calculated from dose response curves, which were obtained from titration experiments (n = 4). Data are expressed as mean±SD.
