# Supplementary material for: MicroRNA-100-5p and microRNA-298-5p released from apoptotic cortical neurons are endogenous Toll-like receptor 7/8 ligands that contribute to neurodegeneration
Source: Mol Neurodegener. 2021 Nov 27;16:80. doi: 10.1186/s13024-021-00498-5 (PMC8626928; doi:10.1186/s13024-021-00498-5)
Supplement: Supplementary file 8 — Additional file 8. let-7g-5p and miR-672-5p induce neuronal injury in vitro. (a, b) Co-cultures of C57BL/6 (wild-type, WT) microglia and neurons were incubated with 5 μg/ml of indicated miRNAs for 5 d. Mutant control oligonucleotide and unstimulated cells were used as negative control. Cells were subsequently immunostained with NeuN antibody and stained with TUNEL assay and DAPI. Quantification of NeuN (depicted as relative neuronal viability, left)- and TUNEL (right)-positive cells in co-cultures. Data are expressed as mean ± SD, n = 4. *P < 0.05; **P < 0.01; ***P < 0.001 compared to unstimulated condition, Student’s t-test. (c) Enriched WT and Tlr7−/− cortical neurons were incubated with 5 μg/ml of indicated miRNAs for 5 d. Cell cultures were subsequently immunostained with NeuN antibody and with DAPI. LPS (100 ng/ml) was used to test for potential relevant contamination of enriched neuronal cell cultures with microglia. Mutant control oligonucleotide and unstimulated cells were used as negative control. NeuN-positive neurons were quantified, and data are depicted as relative neuronal viability of cells treated with the indicated miRNA compared to control. Results are shown as mean ± SD (n = 4 for WT, n = 3 for Tlr7−/− neurons). P value as indicated compared to unstimulated condition, Student’s t-test. [file 13024_2021_498_MOESM8_ESM.pdf]

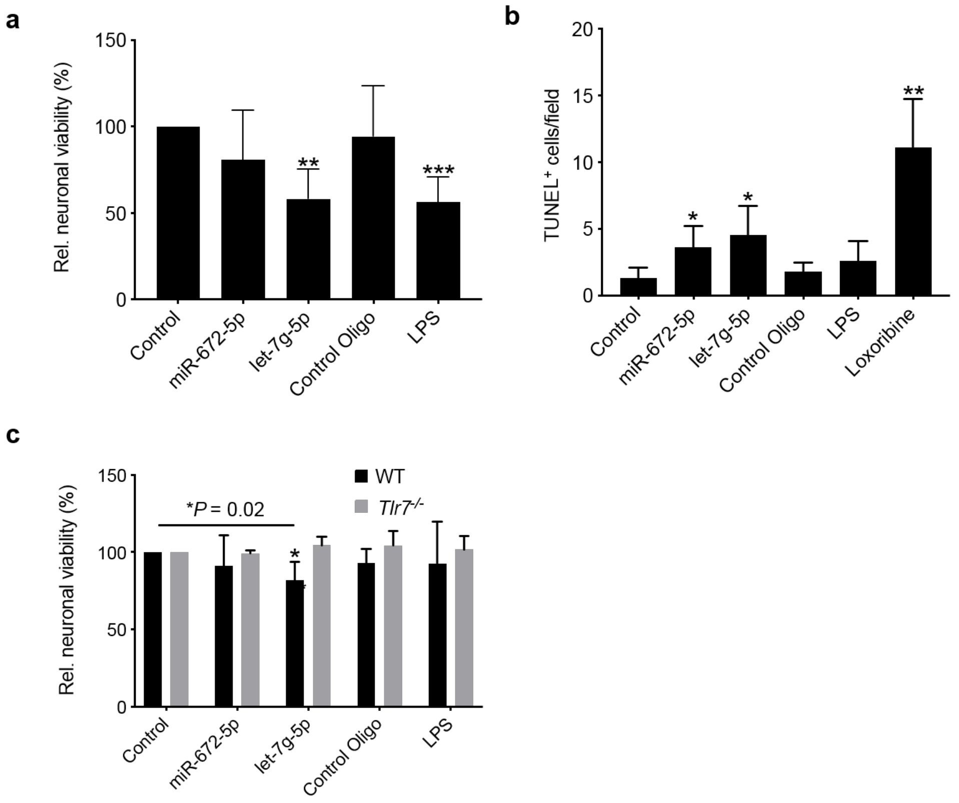

Additional file 8 let-7g-5p and miR-672-5p induce neuronal injury *in vitro*. (**a**, **b**) Co-cultures of C57BL/6 (wild-type, WT) microglia and neurons were incubated with 5 µg/ml of indicated miRNAs for 5 d. Mutant control oligonucleotide and unstimulated cells were used as negative control. Cells were subsequently immunostained with NeuN antibody and stained with TUNEL assay and DAPI. Quantification of NeuN (depicted as relative neuronal viability, left)- and TUNEL (right)-positive cells in co-cultures. Data are expressed as mean±SD, n = 4. *$P < 0.05$; **$P < 0.01$; ***$P < 0.001$ compared to unstimulated condition, Student's t-test. (**c**) Enriched WT and $Tlr7^{-/-}$ cortical neurons were incubated with 5 µg/ml of indicated miRNAs for 5 d. Cell cultures were subsequently immunostained with NeuN antibody and with DAPI. LPS (100 ng/ml) was used to test for potential relevant contamination of enriched neuronal cell cultures with microglia. Mutant control oligonucleotide and unstimulated

cells were used as negative control. NeuN-positive neurons were quantified, and data are depicted as relative neuronal viability of cells treated with the indicated miRNA compared to control. Results are shown as mean±SD (*n* = 4 for WT, *n* = 3 for *Tlr7*$^{-/-}$ neurons). *P* value as indicated compared to unstimulated condition, Student's *t*-test.
