# Supplementary material for: MicroRNA-100-5p and microRNA-298-5p released from apoptotic cortical neurons are endogenous Toll-like receptor 7/8 ligands that contribute to neurodegeneration
Source: Mol Neurodegener. 2021 Nov 27;16:80. doi: 10.1186/s13024-021-00498-5 (PMC8626928; doi:10.1186/s13024-021-00498-5)
Supplement: Supplementary file 9 — Additional file 9. miR-298-5p and miR-100-5p enter neurons and co-localize to their endosomal compartment and TLR7. (a) Enriched C57BL/6 cortical neurons were incubated with 40 μg/ml pHrodo Red Dextran serving as endosomal marker for 20 min. Subsequently, neurons were exposed to 5 μg/ml of Alexa488-labeled miR-298-5p or Alexa488-labeled miR-100-5p, and fixed after 4 h. Scale bar, 10 μm. (b) Neurons exposed to the fluorescence-tagged miRNAs, as described above, were fixed and immunolabeled with TLR7 antibody. Scale bar, 20 μm. (a, b) Cells were analyzed by confocal microscopy with sequential analysis. Representative images of neurons incubated with the indicated fluorescent miRNAs (488 nm, green) and pHrodo Red Dextran or TLR7 (552 nm, red) are shown (left panel). Diagrams depict fluorescence intensities of the marked ROI in neurons for the sequential analysis used (pHrodo Red Dextran/TLR7: red line; fluorescent miRNA: green line, right panel). [file 13024_2021_498_MOESM9_ESM.pdf]

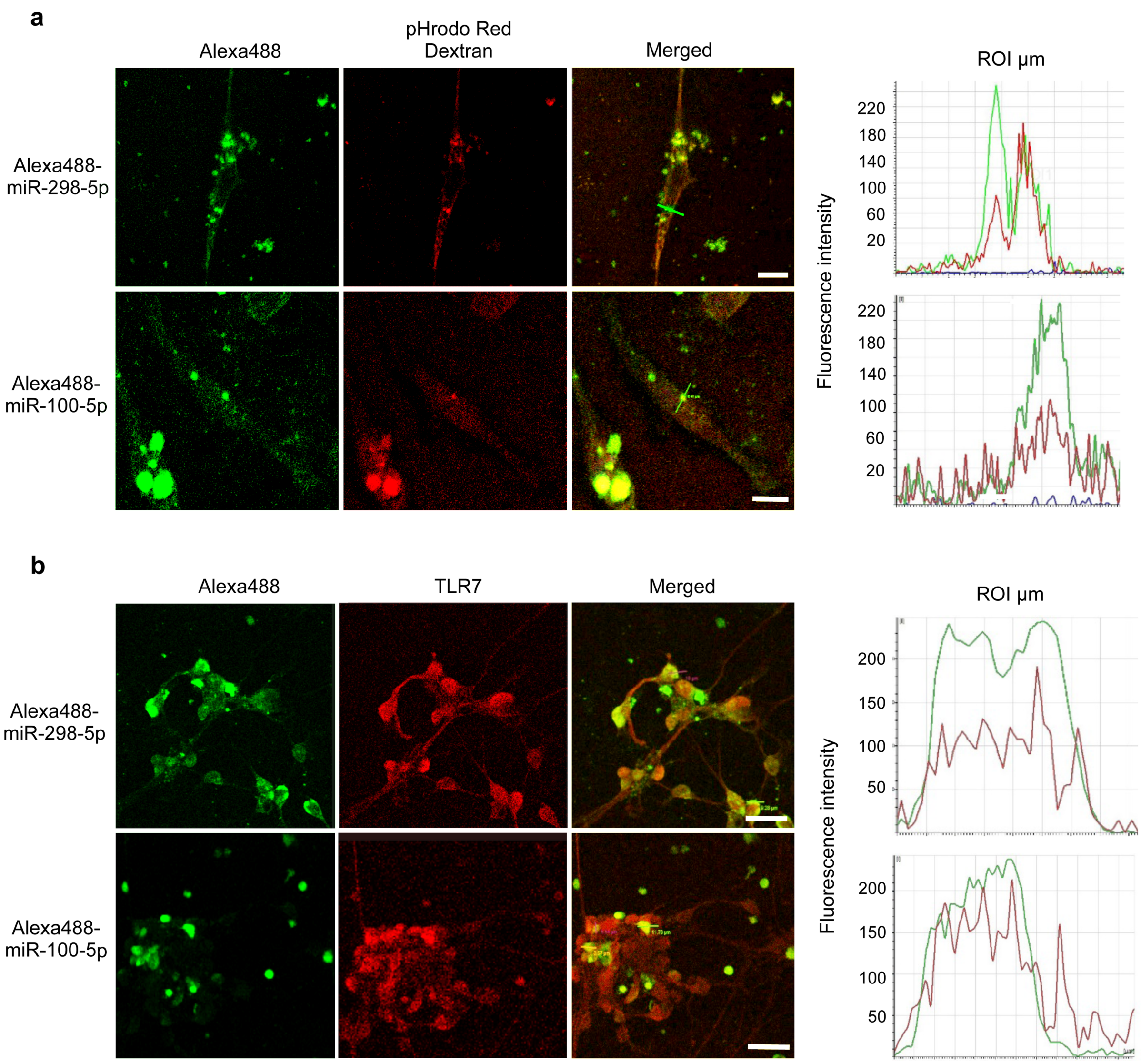

Additional file 9 miR-298-5p and miR-100-5p enter neurons and co-localize to their endosomal compartment and TLR7. (**a**) Enriched C57BL/6 cortical neurons were incubated with 40 µg/ml pHrodo Red Dextran serving as endosomal marker for 20 min. Subsequently, neurons were exposed to 5 µg/ml of Alexa488-labeled miR-298-5p or Alexa488-labeled miR-100-5p, and fixed after 4 h. Scale bar, 10 µm. (**b**) Neurons exposed to the fluorescence-tagged miRNAs, as described above, were fixed and immunolabeled with TLR7 antibody. Scale bar, 20 µm. (**a**, **b**) Cells were analyzed by confocal microscopy with sequential analysis. Representative images of neurons incubated with the indicated fluorescent miRNAs (488 nm, green) and

pHrodo Red Dextran or TLR7 (552 nm, red) are shown (left panel). Diagrams depict fluorescence intensities of the marked ROI in neurons for the sequential analysis used (pHrodo Red Dextran/TLR7: red line; fluorescent miRNA: green line, right panel).
