# Supplementary material for: MicroRNA-100-5p and microRNA-298-5p released from apoptotic cortical neurons are endogenous Toll-like receptor 7/8 ligands that contribute to neurodegeneration
Source: Mol Neurodegener. 2021 Nov 27;16:80. doi: 10.1186/s13024-021-00498-5 (PMC8626928; doi:10.1186/s13024-021-00498-5)
Supplement: Supplementary file 10 — Additional file 10. Intrathecal brain-derived, small RNA triggers neurodegeneration. 125 pmol of an LNA inhibitor specific for mmu-miR-298-5p (miR-298-I), an LNA negative control inhibitor (neg. co.-I), or solvent (H2O) were injected intrathecally into C57BL/6 mice. After 16 h, mice were injected intrathecally for a second time with 10 μg of small RNA enriched from C57BL/6 mouse brain RNA, or solvent (solvent + solvent (sham), n = 4; solvent + small RNA (smRNA), n = 4; miR-298-5p inhibitor + small RNA (miR-298-I + smRNA), n = 4; neg. control inhibitor + small RNA (neg. co.-I + smRNA), n = 4). After a further 3 d, brain sections were immunolabeled with a NeuN antibody, and with DAPI. (a) Representative images of brain sections labeled with NeuN antibody and DAPI are shown. Scale bar, 50 μm; insets, scale bar, 10 μm. (b) NeuN+ cells in the cerebral cortex were quantified. Data are shown as mean ± SD. P values for relevant groups as determined using the Student’s t-test are shown. n.s., not significant. [file 13024_2021_498_MOESM10_ESM.pdf]

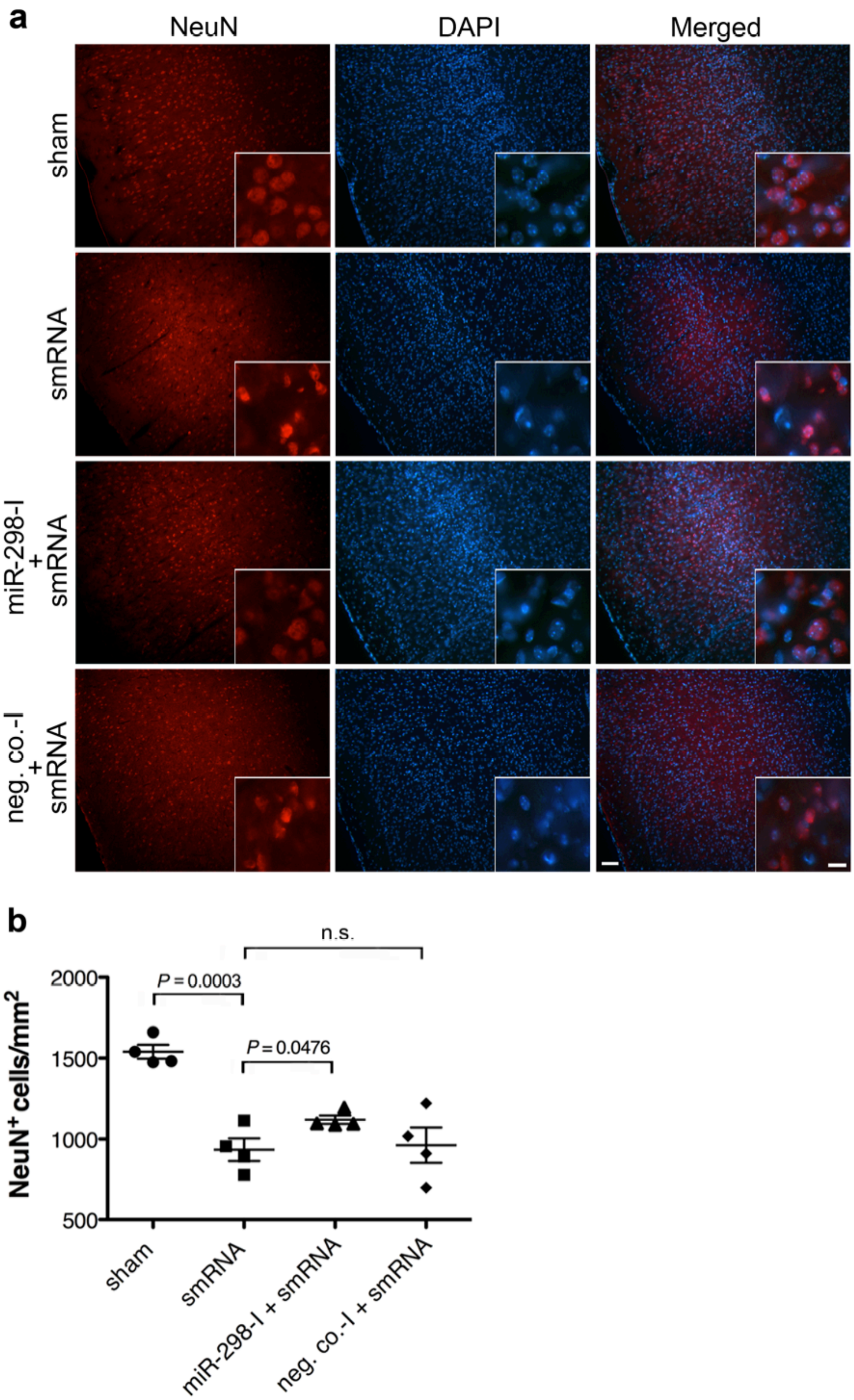

Additional file 10 Intrathecal brain-derived, small RNA triggers neurodegeneration. 125 pmol of an LNA inhibitor specific for mmu-miR-298-5p (miR-298-I), an LNA

negative control inhibitor (neg. co.-I), or solvent (H_2O) were injected intrathecally into C57BL/6 mice. After 16 h, mice were injected intrathecally for a second time with 10 µg of small RNA enriched from C57BL/6 mouse brain RNA, or solvent (solvent + solvent (sham), *n* = 4; solvent + small RNA (smRNA), *n* = 4; miR-298-5p inhibitor + small RNA (miR-298-I + smRNA), *n* = 4; neg. control inhibitor + small RNA (neg. co.-I + smRNA), *n* = 4). After a further 3 d, brain sections were immunolabeled with a NeuN antibody, and with DAPI. (**a**) Representative images of brain sections labeled with NeuN antibody and DAPI are shown. Scale bar, 50 μm; insets, scale bar, 10 μm. (**b**) $NeuN^+$ cells in the cerebral cortex were quantified. Data are shown as mean±SD. *P* values for relevant groups as determined using the Student's *t*-test are shown. n.s., not significant.
