# Supplementary material for: MicroRNA-100-5p and microRNA-298-5p released from apoptotic cortical neurons are endogenous Toll-like receptor 7/8 ligands that contribute to neurodegeneration
Source: Mol Neurodegener. 2021 Nov 27;16:80. doi: 10.1186/s13024-021-00498-5 (PMC8626928; doi:10.1186/s13024-021-00498-5)
Supplement: Supplementary file 11 — Additional file 11. Intrathecal pre-treatment with LPS enhances miR-298-5p- and miR-100-5p-induced neurodegeneration and microglial accumulation. 1 μg LPS was injected intrathecally into C57BL/6 mice. After 16 h, mice were injected intrathecally with 10 μg of miR-298-5p, miR-100-5p, or control oligoribonucleotide (LPS alone, n = 4; LPS + control oligo, n = 4; LPS + miR-298-5p, n = 4; LPS + miR-100-5p, n = 4). Naive mice and mice solely injected with control oligoribonucleotide, miR-100-5p, or miR-298-5p were included in this experimental set-up (naive, n = 4; control oligo, n = 4; miR-298-5p, n = 4; miR-100-5p, n = 4). After 3 d, brain sections were immunostained with NeuN or Iba1 antibody, and with DAPI. Representative images of brain sections labeled with NeuN antibody (a), Iba1 antibody (c), and DAPI (a, c) are shown. Scale bar, 50 μm; inserts, scale bar, 10 μm. NeuN+ (b) and Iba1+ (d) cells in the cerebral cortex were quantified. Data are shown as mean ± SD. P values for relevant groups as determined using the Student’s t-test are shown in (b) and (d). n.s., not significant. [file 13024_2021_498_MOESM11_ESM.pdf]

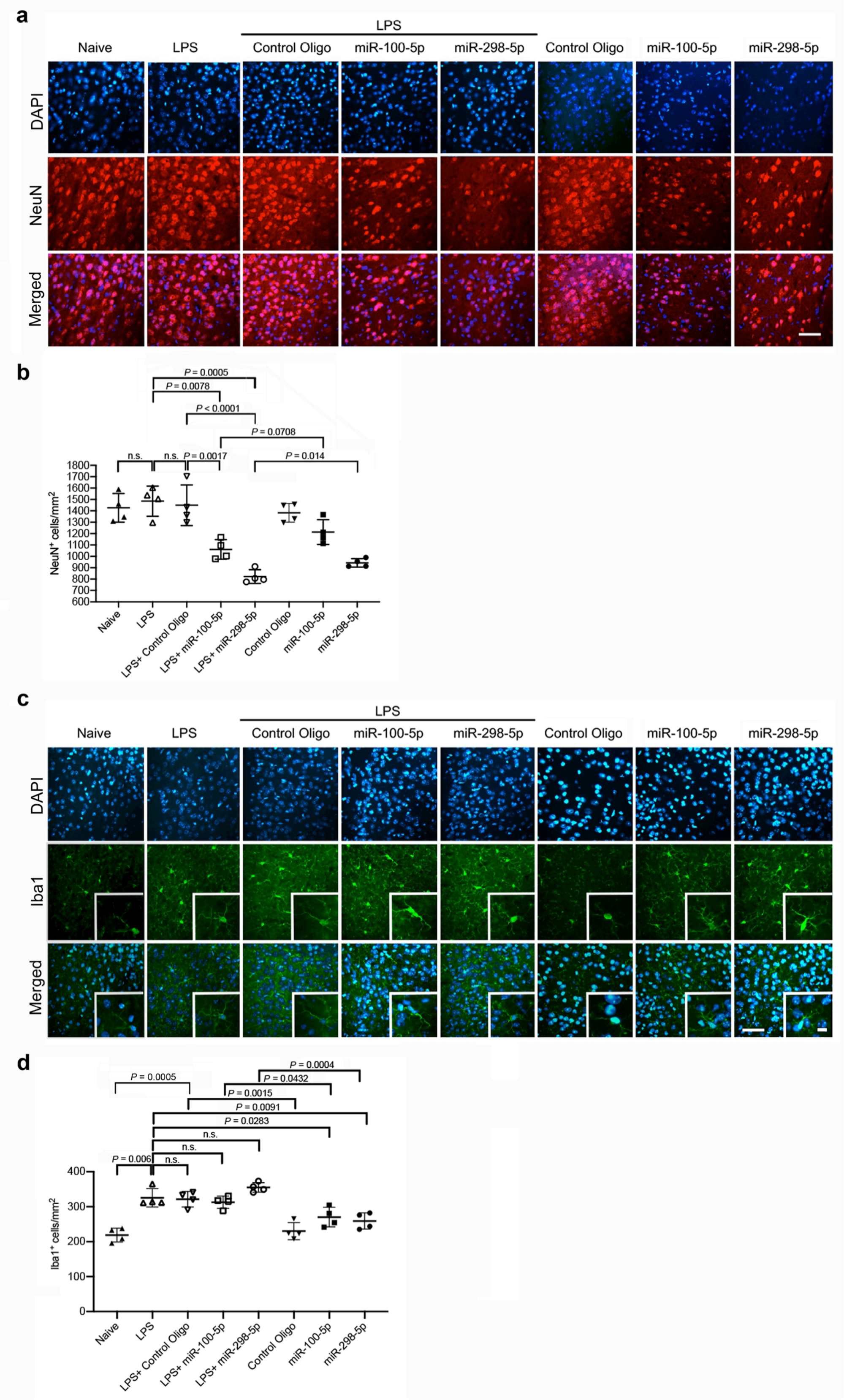


**Additional file 11** Intrathecal pre-treatment with LPS enhances miR-298-5p- and miR-100-5p-induced neurodegeneration and microglial accumulation. 1 µg LPS was

injected intrathecally into C57BL/6 mice. After 16 h, mice were injected intrathecally with 10 µg of miR-298-5p, miR-100-5p, or control oligoribonucleotide (LPS alone, *n* = 4; LPS + control oligo, *n* = 4; LPS + miR-298-5p, *n* = 4; LPS + miR-100-5p, *n* = 4). Naive mice and mice solely injected with control oligoribonucleotide, miR-100-5p, or miR-298-5p were included in this experimental set-up (naive, *n* = 4; control oligo, *n* = 4; miR-298-5p, *n* = 4; miR-100-5p, *n* = 4). After 3 d, brain sections were immunostained with NeuN or Iba1 antibody, and with DAPI. Representative images of brain sections labeled with NeuN antibody (**a**), Iba1 antibody (**c**), and DAPI (**a**, **c**) are shown. Scale bar, 50 μm; inserts, scale bar, 10 μm. $NeuN^+$ (**b**) and $Iba1^+$ (**d**) cells in the cerebral cortex were quantified. Data are shown as mean±SD. *P* values for relevant groups as determined using the Student's *t*-test are shown in (**b**) and (**d**). n.s., not significant.
