# Supplementary material for: MicroRNA-100-5p and microRNA-298-5p released from apoptotic cortical neurons are endogenous Toll-like receptor 7/8 ligands that contribute to neurodegeneration
Source: Mol Neurodegener. 2021 Nov 27;16:80. doi: 10.1186/s13024-021-00498-5 (PMC8626928; doi:10.1186/s13024-021-00498-5)
Supplement: Supplementary file 13 — Additional file 13: Table S2. miRNA concentrations used in this study given in [nm]. [file 13024_2021_498_MOESM13_ESM.docx]

|  |  | **Concentration in nM for** | | | | |
| --- | --- | --- | --- | --- | --- | --- |
| **miRNA** | **Molecular weight g/mol** | **20 µg/ml** | **10 µg/ml** | **5 µg/ml** | **1 µg/ml** | **0.1 µg/ml** |
| miR-151-5p | 7113.3 | 2811.63 | 1405.82 | 702.91 | 140.58 | 14.06 |
| miR-672-5p | 7871.7 | 2540.75 | 1270.37 | 635.19 | 127.04 | 12.70 |
| miR-674-5p | 7594.6 | 2633.45 | 1316.73 | 658.36 | 131.67 | 13.17 |
| miR-361-5p | 7419.5 | 2695.60 | 1347.80 | 673.90 | 134.78 | 13.48 |
| let-7g-5p | 7478.5 | 2674.33 | 1337.17 | 668.58 | 133.72 | 13.37 |
| miR-652-3p | 7169.3 | 2789.67 | 1394.84 | 697.42 | 139.48 | 13.95 |
| miR-342-5p | 7532.5 | 2655.16 | 1327.58 | 663.79 | 132.76 | 13.28 |
| miR-100-5p | 7418.5 | 2695.96 | 1347.98 | 673.99 | 134.80 | 13.48 |
| miR-7020-5p | 8356.2 | 2393.43 | 1196.72 | 598.36 | 119.67 | 11.97 |
| miR-128-3p | 7011.2 | 2852.58 | 1426.29 | 713.14 | 142.63 | 14.26 |
| miR-298-5p | 7827.8 | 2555.00 | 1277.50 | 638.75 | 127.75 | 12.77 |
| miR-501-3p | 7497.6 | 2667.52 | 1333.76 | 666.88 | 133.38 | 13.34 |

**Additional table 2.** Conversion of concentrations from [µg/ml] into [nM].
